# A preclinical pipeline to evaluate migrastatics as therapeutic agents in metastatic melanoma

**DOI:** 10.1038/s41416-021-01442-6

**Published:** 2021-06-25

**Authors:** Oscar Maiques, Bruce Fanshawe, Eva Crosas-Molist, Irene Rodriguez-Hernandez, Alessia Volpe, Gaia Cantelli, Lena Boehme, Jose L. Orgaz, Faraz K. Mardakheh, Victoria Sanz-Moreno, Gilbert O. Fruhwirth

**Affiliations:** 1 Centre for Tumour Microenvironment at Barts Cancer Institute, Queen Mary University of London, Charterhouse Square Campus, John Vane Science Centre, London, UK; 2 Randall Division of Cell and Molecular Biophysics, King’s College London, New Hunt’s House, Guy’s Campus, London, UK; 3 Imaging Therapies and Cancer Group, Comprehensive Cancer Centre, School of Cancer and Pharmaceutical Studies, King’s College London, Guy’s Campus, London, UK; 4 Centre for Cancer Cell & Molecular Biology at Barts Cancer Institute, Queen Mary University of London, Charterhouse Square Campus, John Vane Science Centre, London, UK; 5 School of Biomedical Engineering and Imaging Sciences, King’s College London, St. Thomas’ Hospital, London, UK; 6 Present Address: Molecular Imaging Group, Department of Radiology, Memorial Sloan Kettering Cancer Center, New York, NY USA; 7 Present Address: Instituto de Investigaciones Biomedicas ‘Alberto Sols’, CSIC-UAM, Madrid, Spain

**Keywords:** Skin cancer, Cancer models, Drug discovery, Metastasis

## Abstract

**Background:**

Metastasis is a hallmark of cancer and responsible for most cancer deaths. Migrastatics were defined as drugs interfering with all modes of cancer cell invasion and thus cancers’ ability to metastasise. First anti-metastatic treatments have recently been approved.

**Methods:**

We used bioinformatic analyses of publicly available melanoma databases. Experimentally, we performed in vitro target validation (including 2.5D cell morphology analysis and mass spectrometric analysis of RhoA binding partners), developed a new traceable spontaneously metastasising murine melanoma model for in vivo validation, and employed histology (haematoxylin/eosin and phospho-myosin II staining) to confirm drug action in harvested tumour tissues.

**Results:**

Unbiased and targeted bioinformatic analyses identified the Rho kinase (ROCK)-myosin II pathway and its various components as potentially relevant targets in melanoma. In vitro validation demonstrated redundancy of several RhoGEFs upstream of RhoA and confirmed ROCK as a druggable target downstream of RhoA. The anti-metastatic effects of two ROCK inhibitors were demonstrated through in vivo melanoma metastasis tracking and inhibitor effects also confirmed ex vivo by digital pathology.

**Conclusions:**

We proposed a migrastatic drug development pipeline. As part of the pipeline, we provide a new traceable spontaneous melanoma metastasis model for in vivo quantification of metastasis and anti-metastatic effects by non-invasive imaging.

## Background

Metastasis is a hallmark of cancer^1^ but has been difficult to target due to the complexity of its underlying mechanisms and difficulties defining appropriate clinical study end points. Notably, an anti-metastatic prostate cancer drug, apalutamide, was recently approved by the FDA involving the new endpoint of ‘metastasis-free survival’, measuring the length of time that tumours did not spread to other parts of the body or that death occurred after starting treatment.^2^ Recently, we defined a new class of drugs that we coined ‘migrastatics’, and which would comprise drugs interfering with all modes of cancer cell invasion and therefore with their ability to metastasise.^3,4^ Migrastatics have the potential to be of particular value for the treatment of cancers with the propensity to metastasise early. While in most cases metastasis is associated with large primary neoplasms, it can occur in some cancers when primary tumours are still small. Melanoma, which is a particularly aggressive form of skin cancer, belongs to the group of cancers that spread early, with metastasis being reported for lesion sizes below two millimetres.^5^ Moreover, patients suffering from malignant advanced melanoma have a very poor prognosis.^6,7^

Rho kinase (ROCK) downstream of RhoA has been shown to play an important role in tumour growth, cell migration, invasion and survival.^8–10^ The ROCK-myosin II pathway regulates actin polymerisation and cell contractility^11–14^ and is a key regulator of melanoma invasion and metastasis.^9,13,15–17^ Notably, cytoskeletal remodelling and changes in the expression and activity of the ROCK-myosin II pathway components were also recently discovered to play a major role in the acquisition of melanoma resistance to MAPK inhibitors.^18^ Several key regulators of RhoA activity have been described including guanine nucleotide exchange factors (GEFs) and GTPase-activating proteins (GAPs).^19,20^ RhoGEFs have been under extensive investigation as therapeutic targets, for example, various inhibitors of Rho GTPase signalling.^21–23^ Together with ROCK, they represent potential anti-metastatic targets.^3,4^ What is less well understood is which of these molecules would be an effective target. More generally, development of migrastatic drugs is dependent on refinement of approval regulations as well as widespread screening for compounds with low general toxicity but high efficacy against cancer cell spread as determined in 3D cell models and pre-clinical animal models. The latter are required to reproduce the metastatic process as closely as possible to the human scenario. Preclinical metastasis models would greatly benefit from strategies to visualise cancer cells in vivo and quantitatively track their spread over time to assess the efficacy of migrastatics on the preclinical level.

Long-term observation and quantification of cancer and its spread is achievable in pre-clinical models by non-invasive reporter gene-based radionuclide imaging. We have previously demonstrated that whole-body medical imaging technologies such as single-photon emission computed tomography (SPECT) or positron emission tomography (PET) in combination with reporter gene technology and appropriate imaging probes enabled quantitative in vivo tracking of murine cancer and its metastasis.^24–27^ Therefore, we employed the sodium iodide symporter (NIS) as a radionuclide reporter. To streamline the generation of in vivo traceable cancer cell lines and their ex vivo detection (*e.g*. by histology or flow cytometry) we combined the radionuclide reporter NIS with a fluorescent protein such as GFP or RFP.^26,27^

Here, we demonstrate a systematic ‘pipeline’ approach for the preclinical development of migrastatics, which includes unbiased and targeted bioinformatic analyses for target identification, relevant examples for in vitro target validation, a specifically and newly developed traceable and spontaneously spreading melanoma model to quantify metastasis in vivo as well as the tissue-level validation of candidate migrastatic efficacy.

## Methods

Information regarding cell proliferation and cellular radiotracer uptake assays, flow cytometry and microscopy of cells, immunoblotting and radioactivity analysis in tissues is detailed in Supplementary Information (SI).

### Reagents

All standard chemicals and molecular biology reagents were either from Fisher-Scientific, NEB, Millipore, Sigma or VWR. Tissue culture materials were from Corning, Sarstedt or TPP. Tc-99m-pertechnetate ([^99m^Tc]TcO_4_^−^) in saline was eluted from a Drytec generator (GE Healthcare, UK) from the Radiopharmacy at Guy’s and St. Thomas’ Hospital Nuclear Medicine Department. Wheat germ agglutinin (WGA) conjugated to AlexaFluor488 was from Invitrogen. Mowiol was from ICN (Costa Mesa, CA, US). ROCK inhibitors: Y27632 (Tocris Bioscience, UK), GSK269962 (Axon Medchem, Netherlands), H1152 (Merck, Germany), AT13148 (Selleckchem, USA). SiRNAs targeting ARHGEF1, ARHGEF11 and ARHGEF2 were from Dharmacon (siGENOME SMARTpool). Primary antibodies: polyclonal rabbit anti-mCherry (Abcam; #ab167543; 1.0 µg/mL for immunoblotting, 3.3 µg/mL for immunohistochemistry), polyclonal rabbit anti-phospho myosin light chain specific for phosphorylation at residue Ser19 (pMLC2) (Cell Signalling Technologies (CST); #3671; 10 µg/mL for immunohistochemistry), monoclonal mouse anti-GAPDH (Genetex; #GTX239; 0.3 µg/mL for immunoblotting), monoclonal mouse anti-MLC (MYL9/MYL12A/B; clone E-4; Santa Cruz Biotechnology; 0.4 µg/mL for immunoblotting), monoclonal rabbit anti-ARHGEF1 (clone D25D2; CST; #D3669, 1:1000 dilution for immunoblotting) and anti-ARHGEF2 (clone 55B6; CST; #4076, 1:1000 dilution for immunoblotting), mouse monoclonal anti-ARHGEF11 (Santacruz Biotechnology; clone D-9; 0.4 µg/mL for immunoblotting). Secondary antibodies: for immunofluorescence staining a Cy5-conjugated goat anti-rabbit antibody (Jackson Immunoresearch; #111-175-144; 2.0 µg/mL) was used, while for immunoblotting ECL Plus or Prime ECL detection systems (GE Healthcare) with horseradish peroxidase (HRP)-conjugated secondary antibodies (GE Healthcare) were used for detection. For immunohistochemistry, relevant secondary antibodies were conjugated to HRP and included polyclonal IgG-HRP specific for either rabbit or mouse antigens (Dako; #P0448 and #P0447, respectively, each used at 1:100 dilution).

### Cells

4599 murine BRAF^V600E^ melanoma cells were a kind gift from Dr. Amine Sadok (Institute of Cancer Research) and Prof Richard Marais (Cancer Research-UK Manchester Institute). A375M2 human BRAF^V600E^ melanoma cells were a kind gift from R. Hynes (Howard Hughes Medical Institute, MIT, Cambridge, MA, USA^11^). MTLn3E.Δ34 cells were previously described.^26^ 293T cells were purchased from ATCC. All melanoma cell lines were confirmed by STR profiling (DNA Diagnostics Center, Fairfield/OH, USA; May 2016). Melanoma and 293T cells were cultured in DMEM containing 10% (v/v) fetal bovine serum (FBS; Biosera, UK), pyruvate (1 mM) and 4.5 g/L D-glucose. MTLn3E.Δ34 cells were grown in alpha-MEM supplemented with 5% (v/v) FBS. All media were also supplemented with L-glutamine (2 mM), penicillin (100 IU/mL) and streptomycin (50 μg/mL). All cell lines were confirmed negative for mycoplasma (LookOut Mycoplasma PCR Detection Kit, Sigma) quarterly throughout the study.

### Animals

In this study, young adult (5–6 weeks old, 24 ± 1 g) male NOD.Cg-Prkdc^scid^ Il2rg^tm1Wjl^/SzJ mice (NSG; purchased from Charles River UK) were used for all animal experiments. All mice were maintained within the King’s College London Biological Services Unit under specific pathogen-free conditions in a dedicated and licensed air-conditioned animal room (at 23 ± 2 °C and 40–60% relative humidity) under light/dark cycles lasting 12 h every day. They were kept in individually ventilated standard plastic cages (501 cm^2^ floor space; from Tecniplast) including environmental enrichment and bedding material in the form of sterilised wood chips, paper stripes and one cardboard roll per cage. Maximum cage occupancy was five animals, and animals were moved to fresh cages with fresh environmental enrichment and bedding material twice per week. Sterilised tap water and food were available *ad libitum*; food was PicoLab Rodent Diet 20 (LabDiet) in the form of 2.5 × 1.6 × 1.0 cm oval pellets that were supplied at the top of the cages. For imaging, animals were anaesthetised using isoflurane (1.5% (v/v) in pure O_2_). After imaging, mice were either left to recover from anaesthesia (by withdrawal of anaesthetic) in a pre-warmed chamber or sacrificed under anaesthesia by cervical dislocation. Tissues were harvested as indicated in ‘Methods’ sections below and Figure captions. In previous experiments with different traceable cell types expressing NIS as an imaging reporter we showed a clear dependence of the acquired radionuclide imaging signal from the cell number. The standard deviation (SD) of cell pellet signals at identical radiotracer labelling was previously determined to be <30% (at relevant low numbers of <10,000 NIS expressing cells in a pellet of 1 million cells; SD is much lower at higher cell numbers). The detection sensitivity was ~1000 labelled cells/million cells.^26^ This data was used together with an α of 0.05 and a power of ≥90% to determine minimum cohort sizes. For longitudinal experiments, cohort sizes were oversubscribed to hedge against potential adverse effects of metastasis and resultant animal sacrifice, which if premature would endanger the whole study. For initial tumour model validation, 4599.NC and 4599 tumours were grown in cohorts of four animals each. For drug treatment studies, cohort sizes were *N* = 6 with four animals per cohort being subjected to serial imaging. The total number of animals used was 32. No adverse events were associated with the procedures performed in this study and animals put on weight in line with strain expectations (data from Charles River UK) throughout. Sentinel animals were kept on the same IVC racks as experimental animals and confirmed to be healthy after completion of the studies.

### Melanoma cell line and patient mRNA database analysis

Expression data of primary melanocytes and melanoma cell lines were downloaded from public database websites and normalised as previously described.^28,29^ Briefly, we used four melanocyte datasets from ref. ^28^ (GSE4570, GSE4840), refs. ^30,31^, and data from melanoma cell lines (Mannheim cohort GSE4843 and Philadelphia cohort GSE4841) were obtained from ref. ^28^ Expression data from human melanoma patient studies; GSE8401,^32^ GSE7553,^33^ GSE3189,^34^ and GSE46517^35^ were extracted from Gene Expression Omnibus (GEO) and normalised using Gene Pattern software (http://www.broadinstitute.org/cancer/software/genepattern/). Normalised mRNA gene expression of 389 human melanoma samples and mutational data of 311 human melanoma samples from TCGA (The Cancer Genome Atlas) database were downloaded from cBioportal.^36,37^ We only considered TCGA samples with greater than 70% tumour cell content from patients who had not received neo-adjuvant treatment prior to tumour resection.

### REACTOME pathway analysis

Genes significantly upregulated and downregulated in metastatic versus primary melanomas from the TCGA database were selected using a log2FC ≥ 0.5 and adjusted *p*-value < 0.01. To identify over-represented functional pathways in metastatic versus primary melanomas, these differentially expressed genes were analysed using the WEB-based GEne SeT AnaLysis Toolkit (WebGestalt).^38^ Pathway enrichment was performed using the functional database REACTOME. The parameters for the enrichment analysis included categories with 5–2000 involved genes, the multiple test adjustment used was the Benjamini–Hochberg (BH) method, and the significant enriched pathways were selected using a false discovery rate (FDR) < 0.05. Weighted set cover method was run to reduce redundancy of the gene sets in the enrichment result.

### Differential mass spectrometric analysis of RhoA pulldown

GST-RhoA pulldown of proteins and subsequent mass spectrometric analysis were performed as described previously,^39^ with minor modifications. Briefly, heavy and light SILAC (Stable Isotope Labelling with Amino Acids in Cell Culture)-labelled A375M2 cells were lysed (50 mM Tris-HCl pH 7.5, 150 mM NaCl, 1% NP-40, 10 mM EDTA, plus phosphatase and protease inhibitor cocktails from Roche), and lysates cleared by centrifugation at 8000 × *g* for 20 min. GST pulldowns were performed using purified bacterially-expressed GST (light) or GST-RhoA (heavy) immobilised on glutathione–sepharose beads. 50 µL of the bed volume of beads was added to ∼2 mg of lysates for 1 h, before three 2 mL washes of the beads in lysis buffer, and elution in 50 µL of boiling 2-fold SDS-PAGE sample buffer. The eluates were then mixed and resolved on a 10% Bis-Tris-Midi gel using MOPS buffer (Life Technologies). In-gel trypsin digestions, peptide extractions and liquid-chromatography-coupled tandem mass spectrometry (LC-MS/MS) analysis were performed as described previously.^40^ Mass spectrometry search and quantifications were done by using Maxquant^41^ using the IPI v3.68 database. Hits were selected based on their SILAC ratio value exhibiting a 4-fold or more enrichment in the GST-RhoA vs. GST sample.

### Generation of reporter gene-expressing melanoma cells

4599 melanoma cells were virally transduced to express NIS-mCherry. Lentiviral particles were generated in 293T cells using pLNT SFFV NIS-mCherry, pΔ8.91 and pVSV-G plasmids as described previously.^27^ Cells were expanded and selected based on mCherry fluorescence by FACS sorting using a FACS Aria III (BD Biosciences, UK; 100 μm nozzle, 3 kV, 20kPa). Cells were expanded and grown for one week before reanalysis by flow cytometry. The cell line was considered stable as no change in purity was detected after subsequent culture of four weeks (see Supplementary Materials).

### Animal tumour model

4599.NC cells were trypsinised, washed with pre-warmed Hank’s buffered saline without Ca^2+^ and Mg^2+^ (HBSS), re-suspended in HBSS and counted. Aliquots of 2 × 10^5^ cells in 20 µL HBSS were injected intradermally on the left flank of the mice. Once palpable, tumour volumes were measured with callipers using the formula V = π/6·L·W·D, wherein L is length, W is width and D is depth of the palpable tumour. Tumour volumes were determined by qualified staff using callipers at least every third day throughout the study.

### Drug treatment of tumour-bearing animals

Tumours were established as described above and grown to sizes of ~20 mm^3^, a size reached around 10 days post inoculation. Animals were then randomised and assigned to either vehicle or treatment cohorts before a baseline SPECT/CT scan was performed, and ROCK inhibitor treatment started on the following day. Y27632 was dissolved in phosphate-buffered saline (without Ca^2+^/Mg^2+^; PBS) and delivered intraperitoneally (100 µL) at a dose of 156 µmol/kg every other day to treatment groups, control cohorts received PBS only. GSK269962 (Axon Medchem, Netherlands) was freshly prepared on the day in PBS containing 10% (v/v) Tween-80, 6.5% (v/v) ethanol and 7% (v/v) DMSO as previously described by ref. ^42^ GSK269962 was administered by oral gavage (200 µL) at a dose of 43.5 µmol/kg daily to treatment groups with control cohorts receiving vehicle instead.

### In vivo radionuclide imaging

Mice were anaesthetised using isoflurane (1.5% (v/v) in O_2_) and 20 MBq [^99m^Tc]TcO_4_^−^ in 100 μL HBSS was administered intravenously under anaesthesia. Protective eye gel was applied, and animals were left sedated and placed onto the imaging platform of a nanoSPECT/CT Silver upgrade scanner (Mediso, Hungary). 30 min post radiotracer injection, CT images were acquired (55 kVp tube voltage, 1200 ms exposure time, 360 projections) and 40 min after initial radiotracer administration a static SPECT scan was performed using 1.0 mm collimators (scan duration 30 min). For specificity tests of the NIS radiotracer, animals were first imaged and then rested awake until the radioactivity had decayed sufficiently to be regarded as negligible, i.e., 48 h (0.4% residual ^99m^Tc radioactivity). Subsequently, the competitive substrate perchlorate was administered at a dose of 250 mg/kg and 40 min later animals were re-imaged as described above.

### In vivo image analysis

All SPECT/CT datasets were reconstructed using a Monte Carlo based full 3D iterative algorithm (Tera-Tomo, Mediso, Hungary). Decay correction to time of injection was applied. All images were analysed using VivoQuant software (inviCRO, USA), which enabled the definition of regions of interest (ROIs) in co-registered SPECT/CT images for quantification of radioactivity (SPECT) in tumours and metastases. The total activity in the whole animal (excluding the tail) at time zero was defined as the injected dose (ID). ROIs for different organs were defined to express uptake in each organ as a percentage of injected dose per volume (%ID/mL). The live tumour volume (LTV) was defined as the volume occupied by live tumour cells as NIS expressing tumour cells can only take up radiotracer when viable, because anionic radiotracer uptake requires symport of sodium cations. This process is dependent on an intact Na^+^/K^+^ gradient across the cellular plasma membrane, which is driven by Na^+^/K^+^-ATPase function.^43^ We exploited this NIS feature to determine LTV based on thresholded and background-corrected SPECT signals (using VivoQuant software and its implementation of Otsu’s thresholding^44^).

### Tissue staining and histologic tissue analysis

Formaldehyde-fixed paraffin-embedded (FFPE) tissues were prepared using standard methods and stored for a minimum of 48 h to let radioactivity decay. 5 µm tissue sections were cut using a microtome and adhered to poly-L-lysine slides, dried overnight at 40 °C, de-waxed and subjected to antigen retrieval in a pressure cooker at pH 9.0. Sections were blocked (Dual Endogenous Enzyme Blocking Reagent; Dako, UK) in 1% (w/v) BSA for 60 min at room temperature, incubated with indicated primary antibodies at 4 °C overnight before being stained with a horseradish peroxidase-conjugated secondary antibody (2 µg/mL in TBS) for 60 min at room temperature. Samples were developed using the Liquid DAB + Substrate Chromogen System (Dako, UK) and counterstained with haematoxylin before mounting. Slides were scanned using a Nanozoomer (Hamamatsu, Japan) with images being analysed using QuPath1.02.^45^

Morphologic analysis of tumour tissues was performed on haematoxylin- and eosin-stained sections, with the invasive front (IF) defined as melanoma cells with at least 50% contact with the matrix as previously described.^13,17,46^ Using a 20-fold magnification, cell shape was graded from 0 to 3 (0 = round, 1 = ovoid, 2 = elongated and 3 = spindly), and a cell shape score assigned to IF and tumour body (TB) regions: Cell Shape Score = [(% cells with score ‘0’·0) + (% cells with score ‘1’·1) + (% cells with score ‘2’·2) + (% cells with score ‘3’·3)] as previously described.^13^ The distal invasive front (DIF) was defined as the region separate from the tumour mass that contained individual or small groups of invading tumour cells.

For quantification of pMLC2 levels, using QuPath1.02 positive cell detection was performed, and three different thresholds were applied according to intensity scores (0, 1, 2 and 3). Software was then trained using random trees classification algorithm and combined with intensity information to differentiate tumour from stroma. Based on intensity scores given to cells, an H-score value was extracted from each representative group.

### Statistical analysis

GraphPad Prism v7 (La Jolla, USA) was used to calculate all statistical parameters as indicated. Generally, *p*-values were calculated using significance levels of α = 0.05. In-text numbers indicate means of pooled data ± standard deviation (SD) unless otherwise stated.

## Results

**Scheme 1:**
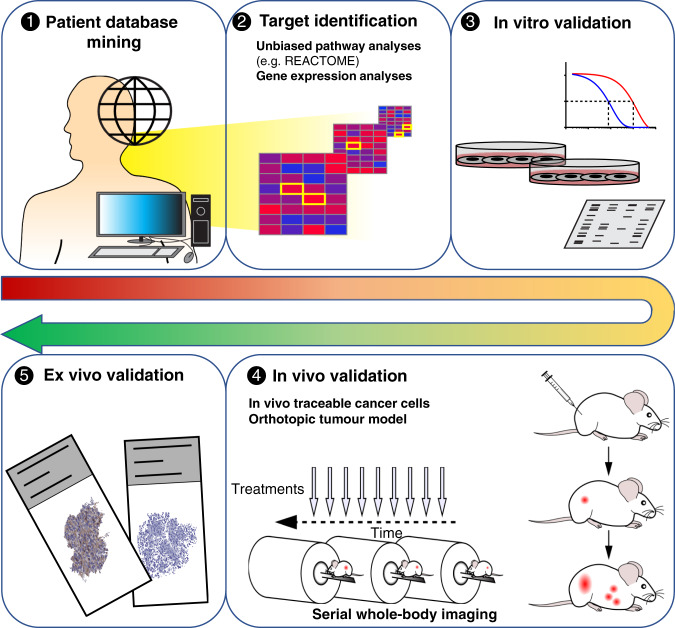
A preclinical pipeline for the development of novel migrastatic drugs. Schematic representation of the main workflow steps performed in this study (numbered to depict the order; for details see text).

We developed a preclinical pipeline to in vitro, in vivo and ex vivo validate candidate anti-metastatic drugs in a spontaneously metastasising non-melanogenic melanoma model (Scheme 1). Individual steps and corresponding results are reported under subsequent sub-headings.

### Database mining for target identification—patient databases revealed upregulation of actomyosin regulators during melanoma progression

**Fig. 1:**
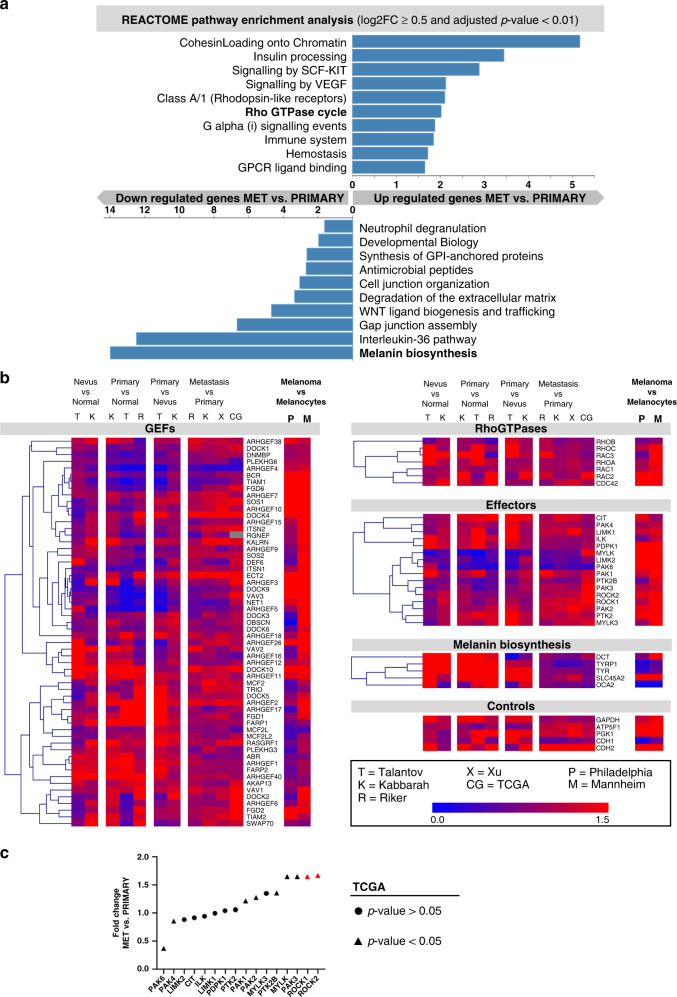
Cancer patient database mining to identify genes involved in melanoma progression. **a** Unbiased REACTOME pathway enrichment analysis of differentially expressed genes in metastatic versus primary melanoma samples from the TCGA database. Analyses were performed using WebGestalt tool, significant pathways were selected using a FDR < 0.05 and bar chart shows enrichment ratios of significant pathways. **b** Using the indicated databases from GEO and TCGA, GEF expression was analysed. Metastatic melanoma, primary melanoma, nevi and normal tissues were compared as indicated. Comparison of melanoma cell lines from both the Philadelphia and Mannheim databases with melanocytes was also performed (right columns). Blue represents downregulation while red represents overexpression between compared groups. Additional expression analyses were performed on RhoGTPases, effector kinases, melanin biosynthesis pathway genes and controls, including ‘house-keeping’ genes and two cadherins. Tabulated numeric data underpinning the shown analyses including *p*-values for each comparison can be found in the Supplementary Tables S1–S5. **c** Fold change expression between metastasis and primary tumours of RhoGTPase effector kinases from TCGA melanoma patients. ROCK1 and ROCK2 highlighted in red, as the most upregulated genes. *P*-values were calculated using unpaired *t*-tests and *t*riangles show statistically significant data (*p* < 0.05).

First, we enquired whether specific cellular signalling pathways were altered when comparing metastatic melanoma lesions with primary melanomas. Therefore, we used gene expression data from The Cancer Genome Atlas (TCGA) and performed pathway enrichment analysis using the open-source curated and peer-reviewed Reactome database implemented in the WEB-based GEne SeT AnaLysis Toolkit (WebGestalt).^38^ This unbiased analysis revealed the significant enrichment of several pathways associated with the up- and downregulated genes when comparing metastatic and primary lesions in melanoma samples (Fig. 1a). The Rho GTPase cycle was among the upregulated signalling pathways, and it has previously been implicated in tumour growth, cell migration and survival.^8–10^ Notably, melanin biosynthesis genes represented the most pronounced downregulated pathway (Fig. 1a).

We next investigated mRNA expression levels of various components belonging to the Rho GTPase cycle and downstream signalling pathways using (i) melanoma patient data from the Gene Expression Omnibus (GEO) studies Talantov, Kabbarah, Riker and Xu, and (ii) TCGA as well as (iii) data stemming from melanocytes and melanoma cell lines including Philadelphia and Mannheim cohorts. We present hierarchical clustering according to expression level changes of 58 GEFs, the major Rho GTPases, and corresponding effector kinases as well as melanin biosynthesis genes (*cf*. above) and five different control genes (Fig. 1b; for tabulated data see Supplementary Tables S1–S5). While there are over 70 human GEFs,^47,48^ our analyses were restricted to 58 GEFs caused by data availability limitations in the microarray-based databases. Expression levels of many Rho-actomyosin contractility regulators were upregulated at various stages of melanoma progression. Notably, increases were more pronounced in metastatic melanoma lesions compared to melanomas at the pre-metastatic stage (Fig. 1b). In contrast, we found melanin biosynthesis genes to be significantly downregulated. We also detected upregulation of CDH2 (neural cadherin) while CDH1 (epithelial cadherin) was downregulated during progression, as was previously reported,^49^ hence both CDH1 and CDH2 data agreed with prior literature reports (Fig. 1b/controls). Moreover, we analysed the previously validated ‘house-keeping’ genes ATP5F1, PGK1 and GAPDH,^50^ which in most comparisons showed little change across datasets. Furthermore, we used the TCGA database and compared RhoGTPases and their effector kinases of metastases and primary tumours. Interestingly, we found that the most upregulated kinases were ROCK1 and ROCK2 (Fig. 1c). We found a significant association between ROCK1/2 expression and NRAS mutations; Fig. S1). These data supported our previous finding that the RhoGTPase cycle was upregulated while melanin biosynthesis genes were downregulated (*cf*. unbiased REACTOME analysis in Fig. 1a).

### Target selection—RhoGEFs act in a redundant manner in metastatic melanoma

**Fig. 2:**
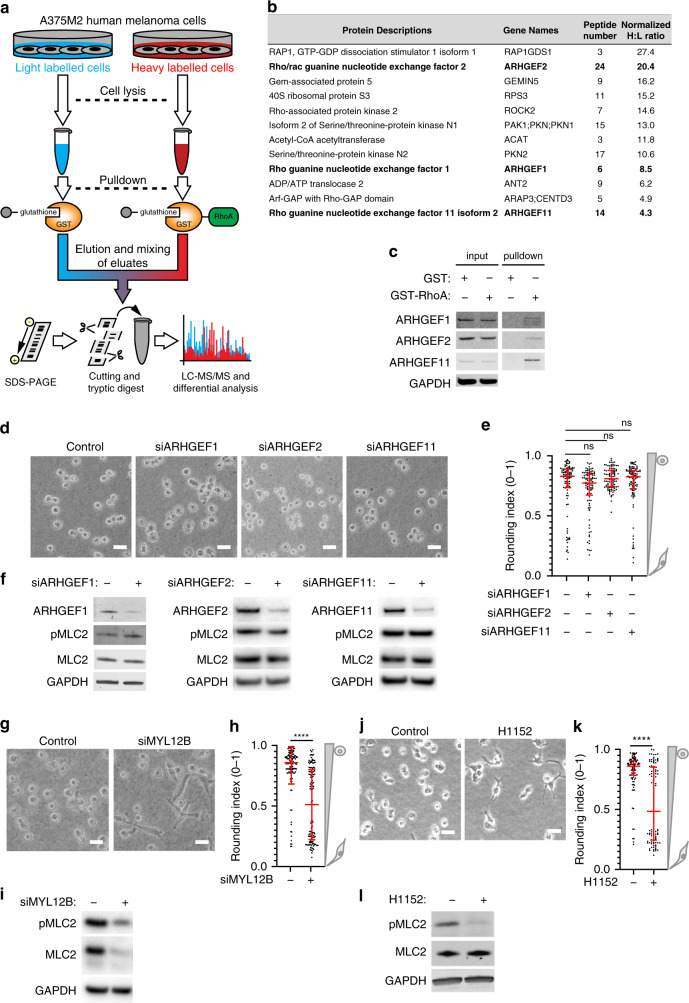
Target identification and validation on the protein level. **a** Experimental schema of the SILAC experiment performed to identify RhoA interacting proteins. Isotope-labelled melanoma cells were lysed followed by incubation with RhoA conjugated to GST-beads or GST-beads alone. Bead samples were subsequently subjected to proteomic analysis by LC-MS/MS. **b** Top hits from the SILAC experiment in (**a**) sorted by the detected heavy:light ratio (H:L) and also listing the number of peptides identified per protein. H:L indicates fold increase in the pulldown of RhoA beads as compared to control beads. **c** Pull-down assay to validate GEFs binding to RhoA detected with SILAC experiment. **d** Representative images for siRNA knock-down of three Rho-GEFs (ARHGEF1, ARHGEF11 and ARHGEF2) in the A375M2 cell line seeded on collagen and **e** the corresponding quantification using the Rounding index (0–1). **f** Immunoblot analyses of pMLC2 and MLC2 levels A375M2 for siRNA knock-down of ARHGEF1, ARHGEF2 and ARHGEF11, respectively. **g** Representative images for siRNA of MYL12B and **h** the corresponding quantification using the Rounding index (0–1). **i** pMLC2 and MLC2 levels in A375M2 cells for siRNA knock-down of MYL12B analysed by immunoblots. **j** Representative images of A375M2 seeded on collagen with or without ROCK inhibition by the inhibitor H1152 and **k** its respective quantification using the Rounding index (0–1). **l** Immunblot analysis of pMLC2 corresponding to experiments in (**j**–**k**). Scale bars in all micrographs are 15 µm. Error bars are SEM and *p*-values were calculated using unpaired *t*-tests; *N* = 3 per cohort. For all panels *p-*values are <0.05 (*), <0.01 (**), <0.001 (***), or <0.0001 (****) and non-significant (ns) as indicated on relevant comparisons.

As we found several RhoGEFs and ROCK1/2 to be increased during melanoma progression (Fig. 1), we first investigated which proteins were binding RhoA in melanoma cells. GTP-bound RhoA binds its key effector ROCK, which in turn regulates myosin II activity via phosphorylation of MLC2. To identify potential activators of RhoA, we employed Stable Isotope Labelling with Amino acids in Cell culture (SILAC) combined with subsequent liquid-chromatography-coupled tandem mass spectrometry analysis. We used the metastatic human melanoma cell line A375M2 that has been used as a model for high ROCK-myosin II pathway activity.^12,13^ GST-RhoA or GST-only were used as baits in pull-down experiments under SILAC conditions with subsequent quantitative proteomic analysis to identify RhoA binders (Fig. 2a). Among the proteins with the highest Heavy (GST-RhoA): Light (GST-only) ratios (Fig. 2b, Supplementary Table S6) were ROCK2 and serine/threonine-protein kinase N2 (PKN2), both well-known effectors of RhoA.^51^ This validated the approach, demonstrating the detection of known RhoA binding partners both upstream and downstream to RhoA. Importantly, we found three different RhoGEFs, which included ARHGEF1, ARHGEF2 and ARHGEF11 (Fig. 2b, Supplementary Table S6). Independent validation of RhoA interactions using separate GST-RhoA pulldown experiments confirmed the results (Fig. 2c).

We next tested whether these RhoGEFs had unique functions impacting actomyosin contractility as a marker for invasive capacity as was previously reported.^13,16,17,46,52^ Using A375M2 amoeboid metastatic melanoma cells seeded on collagen we confirmed that individual RNAi depletion of these three GEFs had no effects on morphology (Fig. 2d, e; cells grown on collagen) and myosin activity (pMLC2; Fig. 2f). In contrast, RNAi depletion of myosin light chain 2 (MYL12B; Fig. 2g–i) and pharmacological inhibition of ROCK1/2 with the small molecule inhibitor H1152 (Fig. 2j–l) resulted in marked reduction of myosin II activity and the loss of the amoeboid ‘rounded’ invasive phenotype on collagen matrices. This data suggested that both regulator of G-protein signalling (RGS) domain-containing RhoGEFs and ARHGEF2 could act redundantly, thereby rendering neither of them a suitable target for full inhibition of actomyosin contractility. Furthermore, these GEFs were not associated with specific melanoma mutations (Fig. S1c-d). In contrast, ROCK1/2 did represent an attractive target in this pathway (*cf*. Fig. 2j–l). These exemplary in vitro validation experiments reduced the number of targets worth investigating in subsequent preclinical in vivo experiments aiming for quantification of potential anti-metastatic effects of drug candidates.

### Generation and characterisation of a new in vivo traceable model of spontaneous melanoma metastasis

**Fig. 3:**
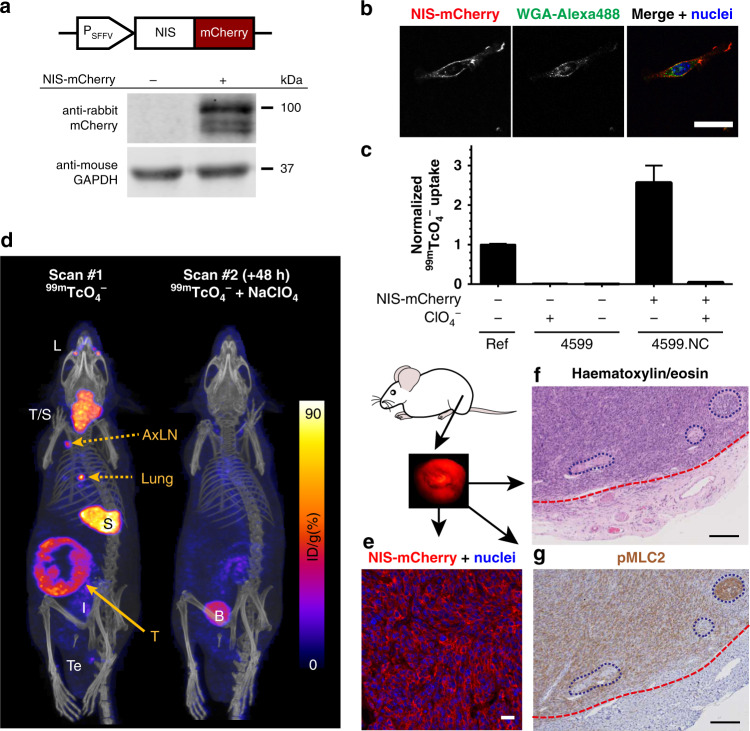
Characterisation of the new in vivo traceable non-melanogenic murine melanoma cell line 4599.NC. **a**/top Sketch of the lentiviral radionuclide-fluorescence fusion reporter gene construct. **a**/bottom Immunoblot analysis of lentivirally transduced and sorted 4599.NC cells compared to parental 4599 cells. **b** Confocal micrographs showing fusion reporter expression and overlap with the plasma membrane stain wheat germ agglutinin (WGA) conjugated to Alexa488. Representative cells are shown; scale bar = 10 µm. **c** Radionuclide reporter function as quantified by uptake of the radioactive NIS substrate [^99m^Tc]TcO_4_^−^. ‘Ref’ indicates a fusion reporter reference cell line as previously described.^26^ Specificity of uptake was demonstrated by abolished radiotracer uptake in the presence of the competitive substrate perchlorate; error bars are SD, *N* = 3. **d** 4599.NC cells were intradermally administered to 5-week-old male NSG mice to establish orthotopic tumours (*N* = 4 animals). Three weeks post administration animals were imaged by [^99m^Tc]TcO_4_^−^-afforded NIS-SPECT/CT clearly indicating cancerous tissues (primary tumour: solid arrow; metastases: dashed arrows) alongside signals stemming from organs expressing NIS endogenously (thyroid and salivary glands (T/G), lachrymal glands (L), stomach (S), and lower in intestine (I) and testes (Te)); none of the latter interfered with the primary tumour or metastases in this model. To assess NIS specificity in vivo, animals received the NIS co-substrate perchlorate intraperitoneally 40 min before animals were re-imaged (48 h after the first imaging session); remaining signals in kidney and bladder (B) reflect radiotracer excretion routes. For corresponding tumour growth curves and ex vivo γ-counting results see Fig. S2. For growth comparison with tumours established from parental 4599 cells see Fig. S2b. **e** Harvested tumour tissues presented with red fluorescence stemming from reporter expression, which not only guided dissection, but enabled histological assessment of tumour tissues. A typical confocal micrograph of one animal from a cohort of *N* = 4 is shown; scale bar = 25 µm. **f** Hematoxilin and eosin staining and (**g**) phospho-MLC2 immunohistochemistry of adjacent tumour sections from the same tumour as in (**e**/**f**); in (**f**/**g**) the red dashed line indicated the tumour front while large blood vessels are encircled with purple dots; scale bars = 200 µm.

To validate inhibitory effects on cancer metastasis in vivo, a reliable spontaneous metastasis model was required. We chose the murine melanoma cell line 4599, which was derived from tumours arising in the *BRAF*^*V600E*^ mouse model^53^ and shown to spontaneously metastasise when transplanted intradermally.^15^ To render 4599 cells traceable in vivo, we engineered them using lentiviral technology to constitutively express the radionuclide reporter NIS fused C-terminally to the red fluorescent protein mCherry (Fig. 3a). We purified reporter-expressing 4599.NIS-mCherry cells (4599.NC) by FACS and confirmed expression of the fusion reporter by immunoblotting (Fig. 3a) and flow cytometry (Fig. S2a). NIS-mCherry co-localised with the plasma membrane marker wheat germ agglutinin (WGA; conjugated to the fluorophore Alexa488), thereby suggesting correct cellular localisation of the reporter (Fig. 3b). NIS function was confirmed by uptake of the radioactive NIS substrate [^99m^Tc]TcO_4_^−^ (Fig. 3c). NIS had not been reported to be expressed in melanoma cells and in line with this, parental 4599 cells did not take up [^99m^Tc]TcO_4_^−^. Specificity of NIS uptake was demonstrated through reduction of [^99m^Tc]TcO_4_^−^ uptake in the presence of the competitive NIS substrate ClO_4_^−^ (Fig. 3c).

4599.NC tumours were established in immunodeficient male NOD.*Cg-Prkdc*^*scid*^*Il2rg*^*tm1Wjl*^/SzJ (NSG) mice. Notably, we found no significant difference in tumour growth between tumours established from parental 4599 and reporter expressing 4599.NC cells (Fig. S2b). In vivo SPECT/CT imaging detected 4599.NC tumours and distant metastases in lung and lymph nodes (Fig. 3d). Additional signals were detected from organs expressing murine NIS, i.e. thyroid and salivary glands, stomach, lachrymal glands and testes. Signals stemming from the endogenous expression of a host reporter gene could interfere with the detection of cancer cells expressing the reporter. Importantly, such endogenous signals were neither detected in the skin (orthotopic site for melanoma) nor in typical melanoma metastasis target tissues (i.e. lung, lymph nodes, liver, brain, bone were free of endogenous signals). This means the combination of the chosen reporter gene and its use in the context of skin cancer is well suited to study spontaneous metastasis in vivo by non-invasive imaging. We also determined whether the observed signals in cancerous tissues were due to specific NIS uptake or were possibly generated by the enhanced permeability and retention effect (EPR), which is known to play a major role in tumour uptake of various agents delivered via the blood stream.^54^ Animals were therefore re-imaged 2 days after the first imaging session, but with prior administration of the competitive NIS substrate ClO_4_^−^ before [^99m^Tc]TcO_4_^−^ injection to visualise cancer cells. ClO_4_^−^ blocked all NIS-associated signals including all tumour signals demonstrating NIS specificity of [^99m^Tc]TcO_4_^−^ signals in vivo (Fig. 3d: compare left/right panels). Ex vivo analyses of radioactivity in harvested tissues corroborated in vivo imaging data (Fig. S2c). Harvested primary tumour tissues were analysed by histology, whereby 4599.NC cells were readily identified based on reporter presence (Fig. 3e); notably, the reporter also showed the expected plasma membrane localisation. Fig. 3f showed expected tumour morphology (by haematoxylin/eosin staining) and Fig. 3g demonstrated high pMLC2 staining in the same tumour cells. This data demonstrated the suitability of the new 4599.NC melanoma cell line for preclinical in vivo cell tracking studies.

### In vitro target inhibition—selection of candidate ROCK inhibitors for preclinical evaluation

**Fig. 4:**
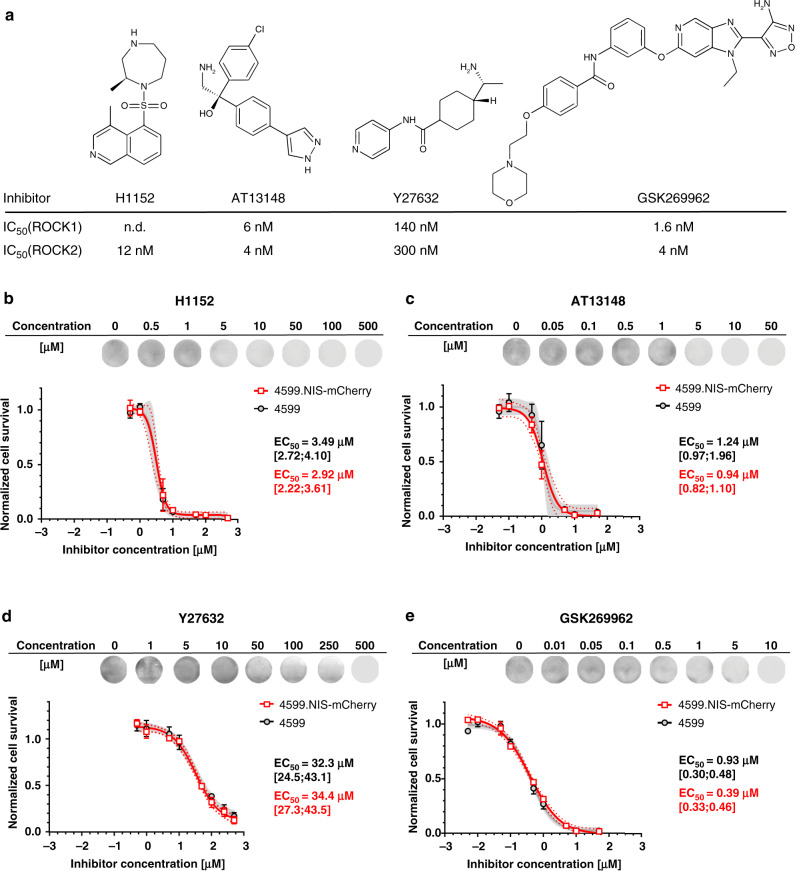
Effects of various ROCK inhibitors on 4599 and 4599.NC melanoma cell survival. **a** Chemical structures and reported IC_50_ values for the listed compounds. Tabled IC_50_ values (nM) were previously reported: H1152^63^; AT13148^64^; Y27632^65^; and GSK269962.^66^ **b**–**e** Comparative cell survival assays in the presence of different concentrations of the indicated ROCK inhibitors revealed no differences between 4599 and 4599.NC cells; *N* = 3, error bars represent SD. EC_50_ including 95% confidence intervals [lower;higher end of range] were calculated using the variable slope dose-response model using Graphpad Prism v7. Grey shades depict 95% confidence intervals for 4599 cells (black circles/grey non-linear fit line) and red dotted lines depict 95% confidence intervals for 4599.NC cells (red squares/red non-linear fit line).

To test whether ROCK1/2 inhibitors impacted on 4599.NC proliferation in vitro, we chose four established ROCK inhibitors: H1152, AT13148, Y27632 and GSK269962^4^ (Fig. 4a). All compounds reduced 4599.NC growth in a comparable manner to parental 4599 cells, but with large differences in efficacy between the different inhibitors (Fig. 4b–e). GSK269962 was most potent, followed by AT13148 and H1152, with Y27632 being the least efficacious compound. We also evaluated the effects of the two more effective compounds (AT13148 and GSK26992A) on the human A375M2 cell model with similar results (Fig. S3). For this exemplary study, Y27632 and GSK269962 were chosen for in vivo experimentation, whereby Y27632 represented a less selective and less potent but frequently used ‘benchmark’ ROCK inhibitor, and GSK269962 represented a highly selective and potent latest-generation ROCK inhibitor.

### Preclinical in vivo imaging to quantify metastasis—in vivo quantification of ROCK inhibitors’ migrastatic properties

**Fig. 5:**
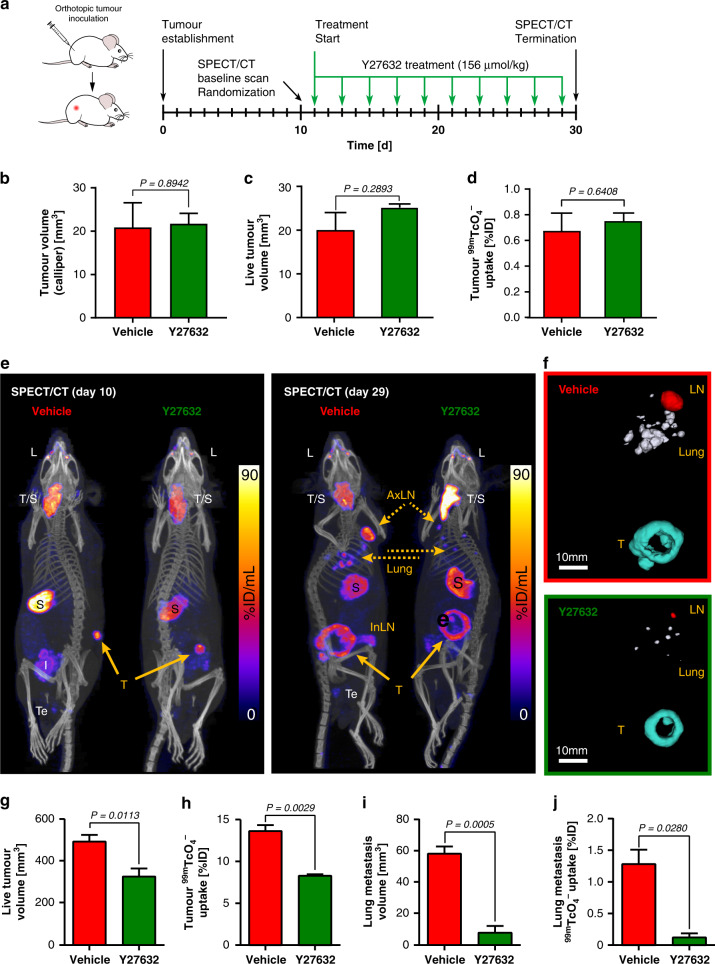
In vivo imaging enables quantification of the migrastatic effect of Y27632. **a** Experimental schema with green arrows indicating intraperitoneal ROCK inhibitor Y27632 administration and black arrows indicate in vivo imaging time points. **b** Tumour volume on day 10 measured using callipers. Data shown are mean tumour sizes of cohorts after animal randomisation. Red represents the future vehicle cohort while green represents the future treatment cohort. Treatments started on the next day, after in vivo SPECT/CT imaging to obtain baseline data. **c** Live tumour volumes (LTV) and **d** radiotracer uptake in tumours at day 10 as determined using SPECT/CT imaging. Colour code as in (**b**). Error bars represent SEM (*N* = 4). **e** Representative maximum intensity projection overlay images of [^99m^Tc]TcO_4_^−^ -afforded NIS-SPECT and CT before treatment start (day 10 ‘baseline’/left) and at the end of the experiment (day 29/right). Right animals in both panels are Y27632-treated while left animals represent control animals. Solid yellow arrows indicated tumours (T) while dotted yellow arrows indicate metastases in lung, axillary lymph nodes (AxLN) and inguinal lymph nodes (InLN)). Organs labelled in white indicate endogenous NIS-expressing organs which did not interfere with sites of metastasis (thyroid and salivary glands (T/S), lachrymal glands (L), stomach (S), low levels in intestine (I) and testes (Te)). **f** 3D volume rendering of live tumour cells based on Otsu-thresholded SPECT signals from (**c**). Vehicle (top) and inhibitor-treated (bottom) boxes contain pseudo-coloured 3D-rendered volumes with tumours visualised in turquoise, lung metastases in grey, and lymph node metastases in red. **g** Live tumour volume (LTV) and **h** radiotracer uptake of tumours was quantified across all animals per cohort. Red represents vehicle- and green ROCK inhibitor-treated animals. Error bars are SEM and *P*-values were calculated using two-tailed unpaired *t*-tests with Welch’s correction; *N* = 4 imaged animals per cohort. **i** LTV and **j** radiotracer uptake in all detected lung metastases combined. Colour code and statistics as in (**g**/**h**).

To quantify the effects of ROCK inhibitors on tumour growth and metastasis we established 4599.NC melanomas by intradermal injection (Fig. 5a). As the focus was on affecting the contractility of tumour cells (and stromal components) but to disentangle it from other potential ROCK inhibitor effects on components of the immune system, we established all tumour models in immunocompromised NSG mice. To model a therapeutic setting, tumours were established in the absence of any treatment and grown until day 10. Tumour-bearing animals were then randomised into control and treatment cohorts; at this point there were no differences between both the cohorts as determined by both calliper measurements and in vivo SPECT/CT imaging (Fig. 5b–d). Subsequently, animals were treated with inhibitors as indicated (Fig. 5a).

After 20 days of treatment, animals were re-imaged by SPECT/CT to determine live tumour volumes (LTV), quantify radiotracer uptake in tumours, and quantify spontaneous metastases (Fig. 5e, f). At this time point, both LTV and radiotracer uptake were significantly reduced in Y27632-treated animals (Fig. 5g, h). Importantly, Y27632 treatment reduced metastasis burden, i.e. LTV of detected secondary lesions in the lungs were significantly reduced (Fig. 5i) as was radiotracer uptake (Fig. 5j). We also investigated whether the latest-generation ROCK inhibitor GSK269962, had a similar effect. Indeed, treatment with GSK269962 resulted in similar results (Fig. S4) compared to Y27632, but they were achieved at lower administered drug doses, which was in line with GSK269962 being a more potent ROCK inhibitor than Y27632 (Fig. 4). Notably, none of the drugs elicited any obvious toxicities at the used doses with animal appearance, behaviour and weight not differing from corresponding vehicle treated animals (Fig. S5).

### Preclinical ex vivo confirmation of ROCK inhibition—effects on the cytoskeleton of melanoma cells

**Fig. 6:**
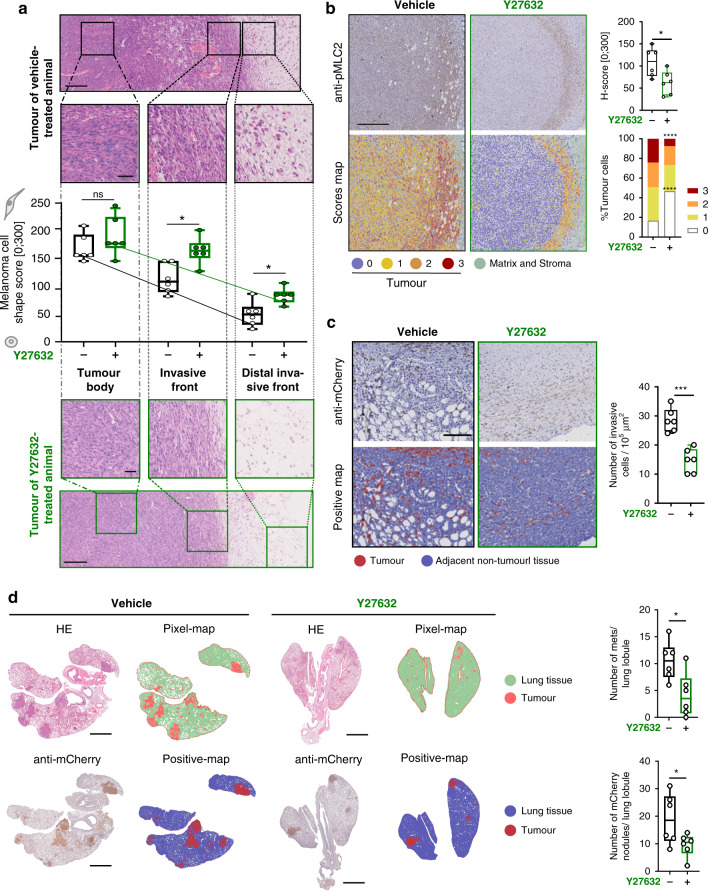
Ex vivo analysis of tumour tissues and lung metastases of animals treated either with Y27632 or vehicle. **a** Haematoxilin- and eosin-stained tumour tissue sections were analysed calculating Cell Shape Scores for regions in the tumour body (TB), the invasive front of the tumour (IF), and for invading cancer cells distant to the invasive front (DIF). Representative tumour sections of vehicle- (top/black) and Y27632-treated (bottom/green) animals with cumulative analysis across all animals (*N* = 6 per cohort) shown in the middle panel. Scale bars are 250 µm in overview images and 50 µm in magnified insets. **b** Histology Score (H-score) for phospho-MLC-stained tumour sections including representative original images (top), score maps (bottom) and cumulative analyses (H-score and %Tumour cells with indicated H-scores) in the right column. Scale bars are 250 µm. **c** Analysis of invading melanoma cells at the DIF. Tumour cells were identified based on immunostaining of the reporter NIS-mCherry using an anti-mCherry antibody (top). Tumour cells were pseudo-coloured (red) in the pixel-positive-map (bottom). Cumulative analyses are shown in the right columns. Scale bar = 100 µm. **d**/left Representative images and pixel-positive maps for haematoxylin and eosin-stained and mCherry-stained lung tissue sections, respectively. **d**/right Corresponding quantitative analyses. Scale bar = 1000 µm. *P*-values were calculated using unpaired *t*-tests and two-way ANOVA with Sidak’s multiple comparison where appropriate. For all panels *P*-values are <0.05 (*), <0.01 (**), <0.001 (***), or <0.0001 (****) as indicated on relevant comparisons.

We first analysed patterns of local invasion in our tumour model. We scored cell morphology and Myosin II levels (pMLC2 staining). Myosin II activity scores from 0 (low) to 3 (very high) were assigned based on p-MLC2 intensity. Cells with very high Myosin II were very abundant in the invasive front (IF) using digital pathology.^45^ Indeed, we observed morphological changes in different areas within a tumour. While melanoma cells were spindly in the tumour body (TB), they underwent cell rounding in the IF (Fig. 6a). Moreover, we defined a further invasive area—the distal invasive front (DIF)—where cancer cells invaded into the dermis (Fig. 6a). Tumour cells at the DIF harboured the highest roundness features observed. Importantly, levels of pMLC2 also progressively increased from TB to IF and IF to DIF (Fig. S6).

Overall, Y26732 treated tumours had decreased Myosin II activity and importantly have reduced the ‘score 3’ population (melanoma cells with highest levels of Myosin II) (Fig. 6b). Importantly, the number of mCherry positive invading cells in the DIF was significantly reduced in Y27632-treated animals (Fig. 6c). Since this tumour model generated spontaneous metastases in lungs (Fig. 3/6), the lungs were also analysed by histology. HE and mCherry-stainings corroborated the in vivo imaging results observed in Fig. 5 as indeed we did observe a significant reduction of lung metastasis burden in mice treated with Y27632 (Fig. 6d).

Taken together, in vivo imaging and ex vivo histology results clearly demonstrated (i) that ROCK inhibitors reached the intended targets, (ii) that there was reduced cell contractility in melanoma cells at the invasive front of tumours, and, importantly, (iii) that ROCK inhibition lowered the number of melanoma cells leaving the primary tumours with the overall consequence of reduced metastasis. This demonstrated the migrastatic efficacies of ROCK inhibitors at the used doses.

## Discussion

Our aim was to demonstrate a systematic ‘pipeline’ approach for the development of migrastatic drugs. We exemplified this pipeline in the context of melanoma, a skin cancer characterised by its high propensity to metastasise.^7^ Unbiased analysis of TCGA data using the REACTOME platform revealed various significant pathway differences between metastatic and primary melanomas including the upregulation of the RhoGTPase cycle and the downregulation of melanin biosynthesis genes (Fig. 1a). This was confirmed through further targeted gene expression analyses of relevant pathway members using available patient datasets (Fig. 1b; Supplementary Tables S1–S5). With the importance of the RhoGEF-Rho-ROCK-myosin II pathway confirmed, we exemplified the target validation process with selected in vitro experiments including comparative mass spectrometry (SILAC), pull-downs and RNAi experiments to assess the role of individual pathway members. Overexpression experiments were avoided due to known caveats of this approach^55^ that had particular relevance for this pathway as many members required tight control and their activity was strongly dependent on cellular localisation.^48,56^ We found that individual knock-down of ARHGEF1/2/11 upstream of RhoA did not yield any phenotypic change, while knocking down MLC2 or using ROCKi appeared a valid strategy (Fig. 2). This data supports using ROCK inhibitors in melanoma to block this pathway downstream of Rho rather than upstream regulators, and builds upon prior work that has shown ROCK inhibition slowing melanoma progression in vivo.^15,16,18,42,57^ For in vivo validation of candidate migrastatics, we specifically developed a new in vivo traceable model of spontaneous melanoma metastasis (Fig. 3). We chose non-melanogenic melanoma, (i) because they lack intrinsically generated melanin as a contrast agent and thus are more challenging to track in vivo; (ii) because our pipeline was intended to serve universally as an example for migrastatic development, and most cancers don’t produce an intrinsic contrast agent such as melanin; and (iii) importantly, we had also found that melanin biosynthesis genes were downregulated during progression (Fig. 1a, b), all of which rendered previously reported melanin-dependent imaging by photoacoustic tomography^58^ unattractive. Consequently, we adopted a different approach, which relied on the proven radionuclide reporter gene NIS.^26,27^ We successfully validated the anti-metastatic actions of two ROCK inhibitors in vivo (Fig. 5, Fig. S4) and confirmed the results ex vivo at the tissue level (Fig. 6, Fig. S6).

Our new in vivo traceable 4599.NC melanoma model enabled 3D quantification of tumour growth and metastatic spread over time (Fig. 3/5, Fig. S4). 4599.NC cells showed [^99m^Tc]TcO_4_^−^ uptake at ~2.5-times higher levels compared to a metastatic breast adenocarcinoma cell line previously used for preclinical metastasis tracking. This suggested 4599.NC cells offered in vivo detectability at least as sensitive as the previously reported reference cells (~1000 cells per million^25,26^). Our work was performed using the radiotracer [^99m^Tc]TcO_4_^−^ suitable for SPECT-CT imaging, but the approach could be readily adapted to potentially more sensitive PET-CT imaging via the NIS PET radiotracer [^18^F]BF_4_^−^. While [^99m^Tc]TcO_4_^−^ is generator-produced and widely available, [^18^F]BF_4_^−^ is now readily available at a high specific activity *via* an automated synthesis protocol.^27^ The chosen reporter gene-based imaging strategy allows unlimited tracking, hence is more appropriate for cancer cell tracking compared to direct cell labelling methods.^59^ We chose a dual-mode radionuclide-fluorescence reporter to streamline model generation and analysis; the radionuclide part NIS of the fusion reporter NIS-mCherry enabled sensitive in vivo cell tracking, while the fluorescence part aided cell line generation and ex vivo tissue analyses. Another important aspect of our non-invasive serial imaging approach is that it reduces the number of animals required for studies with more than one analysis time point. Moreover, repeat imaging of the same animals results in paired data over time, thereby overcoming inter-animal variability and further minimising the number of required animals, which is important to keep the cost of preclinical studies down and limit the number of animals used in drug development. It is also noteworthy that this imaging approach is compatible with co-tracking of therapeutics as we have previously demonstrated using dual-isotope imaging in the context of breast cancer (i.e. co-tracking of cancer and nanomedicine^60^ or cell-based immunotherapy^61^). Similar strategies could be employed using our new traceable melanoma model, too. A transgene mouse that enabled the in vivo tracking of specific cell populations including platelets, T lymphocytes or cardiomyocytes was recently developed.^62^ This reporter mouse could in the future also enable the tracking of specific cancer cell types if crossed with appropriate strains developing spontaneously tumours. However, so far traceable cancer cell models remain restricted to transplantable models such as the one developed here.

Another important aspect of our work is that we demonstrated the actions of the candidate migrastatic drugs at the tissue level as part of this pipeline. The ROCK inhibitors altered the cellular morphology of melanoma cells in line with prior reports and we found the expected reduction of the amoeboid phenotype and Myosin II activity (pMLC2) associated with a reduction of contractility.^12,13,17,18,46^ Amoeboid melanoma cells leaving the tumour bulk and invading through the ECM have been reported previously using intravital imaging (Herraiz, 2015, Sanz-Moreno, 2011). We found that reduced local invasion was accompanied with a decrease in metastatic foci, a phenomenon that has been manifested in our in vivo data by the detection of a lower metastasis burden in such treated animals (Fig. 5).

In conclusion, we present a pipeline approach suitable for the preclinical development of migrastatic drugs. We exemplified this approach in a new in vivo traceable metastatic melanoma model because of melanoma’s high proliferative and particularly invasive phenotype. The methodology described here is readily transferrable to the development of migrastatics for the treatment of other metastatic cancers.

## Supplementary information

Supplementary Materials

## Data Availability

Gene expression datasets re-analysed in this study are available from TCGA and NCBI GEO under accession numbers: GSE4570, GSE4840, GSE4843, GSE4841, GSE23764, GSE8401, GSE7553 and GSE46517.

## References

[1] Hanahan D, Weinberg RA (2011). Hallmarks of cancer: the next generation. Cell.

[2] U.S.Food&DrugAdministration. FDA approves new treatment for a certain type of prostate cancer using novel clinical trial endpoint, https://www.fda.gov/newsevents/newsroom/pressannouncements/ucm596768.htm (2018).

[3] Rosel D, Fernandes M, Sanz-Moreno V, Brabek J (2019). Migrastatics: redirecting R&D in solid cancer towards metastasis?. Trends Cancer.

[4] Gandalovicova A, Rosel D, Fernandes M, Vesely P, Heneberg P, Cermak V (2017). Migrastatics-anti-metastatic and anti-invasion drugs: promises and challenges. Trends Cancer.

[5] Zbytek B, Carlson JA, Granese J, Ross J, Mihm MC, Slominski A (2008). Current concepts of metastasis in melanoma. Expert Rev. Dermatol.

[6] Balch CM, Gershenwald JE, Soong SJ, Thompson JF, Atkins MB, Byrd DR (2009). Final version of 2009 AJCC melanoma staging and classification. J. Clin. Oncol..

[7] Long GV, Menzies AM, Nagrial AM, Haydu LE, Hamilton AL, Mann GJ (2011). Prognostic and clinicopathologic associations of oncogenic BRAF in metastatic melanoma. J. Clin. Oncol..

[8] Rath N, Olson MF (2012). Rho-associated kinases in tumorigenesis: re-considering ROCK inhibition for cancer therapy. EMBO Rep..

[9] Orgaz JL, Herraiz C, Sanz-Moreno V (2014). Rho GTPases modulate malignant transformation of tumor cells. Small GTPases.

[10] Ridley AJ (2015). Rho GTPase signalling in cell migration. Curr. Opin. Cell Biol..

[11] Clark EA, Golub TR, Lander ES, Hynes RO (2000). Genomic analysis of metastasis reveals an essential role for RhoC. Nature.

[12] Sanz-Moreno V, Gadea G, Ahn J, Paterson H, Marra P, Pinner S (2008). Rac activation and inactivation control plasticity of tumor cell movement. Cell.

[13] Sanz-Moreno V, Gaggioli C, Yeo M, Albrengues J, Wallberg F, Viros A (2011). ROCK and JAK1 signaling cooperate to control actomyosin contractility in tumor cells and stroma. Cancer Cell.

[14] Wolf K, Muller R, Borgmann S, Brocker EB, Friedl P (2003). Amoeboid shape change and contact guidance: T-lymphocyte crawling through fibrillar collagen is independent of matrix remodeling by MMPs and other proteases. Blood.

[15] Sadok A, McCarthy A, Caldwell J, Collins I, Garrett MD, Yeo M (2015). Rho kinase inhibitors block melanoma cell migration and inhibit metastasis. Cancer Res.

[16] Georgouli M, Herraiz C, Crosas-Molist E, Fanshawe B, Maiques O, Perdrix A (2019). Regional activation of myosin II in cancer cells drives tumor progression via a secretory cross-talk with the immune microenvironment. Cell.

[17] Cantelli G, Orgaz, Jose L, Rodriguez-Hernandez I, Karagiannis P, Maiques O, Matias-Guiu X (2015). TGF-β-induced transcription sustains amoeboid melanoma migration and dissemination. Curr. Biol..

[18] Orgaz JL, Crosas-Molist E, Sadok A, Perdrix-Rosell A, Maiques O, Rodriguez-Hernandez I (2020). Myosin II reactivation and cytoskeletal remodeling as a hallmark and a vulnerability in melanoma therapy resistance. Cancer Cell.

[19] Bos JL, Rehmann H, Wittinghofer A (2007). GEFs and GAPs: critical elements in the control of small G proteins. Cell.

[20] Sadok A, Marshall CJ (2014). Rho GTPases: masters of cell migration. Small GTPases.

[21] Vigil D, Cherfils J, Rossman KL, Der CJ (2010). Ras superfamily GEFs and GAPs: validated and tractable targets for cancer therapy?. Nat. Rev. Cancer.

[22] Cherfils J, Zeghouf M (2013). Regulation of small GTPases by GEFs, GAPs, and GDIs. Physiol. Rev..

[23] Ferrandez Y, Zhang W, Peurois F, Akendengue L, Blangy A, Zeghouf M (2017). Allosteric inhibition of the guanine nucleotide exchange factor DOCK5 by a small molecule. Sci. Rep..

[24] Ashmore-Harris C, Blackford SJ, Grimsdell B, Kurtys E, Glatz MC, Rashid TS (2019). Reporter gene-engineering of human induced pluripotent stem cells during differentiation renders in vivo traceable hepatocyte-like cells accessible. Stem Cell Res.

[25] Diocou S, Volpe A, Jauregui-Osoro M, Boudjemeline M, Chuamsaamarkkee K, Man F (2017). [(18)F]tetrafluoroborate-PET/CT enables sensitive tumor and metastasis in vivo imaging in a sodium iodide symporter-expressing tumor model. Sci. Rep..

[26] Fruhwirth GO, Diocou S, Blower PJ, Ng T, Mullen GE (2014). A whole-body dual-modality radionuclide optical strategy for preclinical imaging of metastasis and heterogeneous treatment response in different microenvironments. J. Nucl. Med..

[27] Volpe A, Man F, Lim L, Khoshnevisan A, Blower J, Blower PJ (2018). Radionuclide-fluorescence reporter gene imaging to track tumor progression in rodent tumor models. J. Vis. Exp..

[28] Hoek KS, Schlegel NC, Brafford P, Sucker A, Ugurel S, Kumar R (2006). Metastatic potential of melanomas defined by specific gene expression profiles with no BRAF signature. Pigment Cell Res..

[29] Orgaz JL, Ladhani O, Hoek KS, Fernandez-Barral A, Mihic D, Aguilera O (2009). ‘Loss of pigment epithelium-derived factor enables migration, invasion and metastatic spread of human melanoma’. Oncogene.

[30] Ryu B, Kim DS, Deluca AM, Alani RM (2007). Comprehensive expression profiling of tumor cell lines identifies molecular signatures of melanoma progression. PLoS ONE.

[31] Magnoni C, Tenedini E, Ferrari F, Benassi L, Bernardi C, Gualdi G (2007). Transcriptional profiles in melanocytes from clinically unaffected skin distinguish the neoplastic growth pattern in patients with melanoma. Br. J. Dermatol..

[32] Xu L, Shen SS, Hoshida Y, Subramanian A, Ross K, Brunet JP (2008). Gene expression changes in an animal melanoma model correlate with aggressiveness of human melanoma metastases. Mol. Cancer Res..

[33] Riker AI, Enkemann SA, Fodstad O, Liu S, Ren S, Morris C (2008). The gene expression profiles of primary and metastatic melanoma yields a transition point of tumor progression and metastasis. BMC Med. Genomics.

[34] Talantov D, Mazumder A, Yu JX, Briggs T, Jiang Y, Backus J (2005). Novel genes associated with malignant melanoma but not benign melanocytic lesions. Clin. Cancer Res..

[35] Kabbarah O, Nogueira C, Feng B, Nazarian RM, Bosenberg M, Wu M (2010). Integrative genome comparison of primary and metastatic melanomas. PLoS ONE.

[36] Cerami E, Gao J, Dogrusoz U, Gross BE, Sumer SO, Aksoy BA (2012). The cBio cancer genomics portal: an open platform for exploring multidimensional cancer genomics data. Cancer Disco..

[37] Gao J, Aksoy BA, Dogrusoz U, Dresdner G, Gross B, Sumer SO (2013). Integrative analysis of complex cancer genomics and clinical profiles using the cBioPortal. Sci. Signal.

[38] Liao Y, Wang J, Jaehnig EJ, Shi Z, Zhang B (2019). WebGestalt 2019: gene set analysis toolkit with revamped UIs and APIs. Nucleic Acids Res..

[39] Mardakheh FK, Self A, Marshall CJ (2016). RHO binding to FAM65A regulates golgi reorientation during cell migration. J. Cell Sci..

[40] Mardakheh FK, Paul A, Kumper S, Sadok A, Paterson H, McCarthy A (2015). Global analysis of mRNA, translation, and protein localization: local translation is a key regulator of cell protrusions. Dev. Cell.

[41] Cox J, Mann M (2008). MaxQuant enables high peptide identification rates, individualized p.p.b.-range mass accuracies and proteome-wide protein quantification. Nat. Biotechnol..

[42] Vogel CJ, Smit MA, Maddalo G, Possik PA, Sparidans RW, van der Burg SH (2015). Cooperative induction of apoptosis in NRAS mutant melanoma by inhibition of MEK and ROCK. Pigment Cell Melanoma Res..

[43] Dohan O, De la Vieja A, Paroder V, Riedel C, Artani M, Reed M (2003). The sodium/iodide Symporter (NIS): characterization, regulation, and medical significance. Endocr. Rev..

[44] Otsu N (1979). A threshold selection method from gray-level histograms. IEEE Trans. Syst., Man, Cybern..

[45] Bankhead P, Loughrey MB, Fernandez JA, Dombrowski Y, McArt DG, Dunne PD (2017). QuPath: open source software for digital pathology image analysis. Sci. Rep..

[46] Orgaz JL, Pandya P, Dalmeida R, Karagiannis P, Sanchez-Laorden B, Viros A (2014). Diverse matrix metalloproteinase functions regulate cancer amoeboid migration. Nat. Commun..

[47] Goicoechea SM, Awadia S, Garcia-Mata R (2014). I’m coming to GEF you: regulation of RhoGEFs during cell migration. Cell Adh. Migr..

[48] Muller PM, Rademacher J, Bagshaw RD, Wortmann C, Barth C, van Unen J (2020). Systems analysis of RhoGEF and RhoGAP regulatory proteins reveals spatially organized RAC1 signalling from integrin adhesions. Nat. Cell Biol..

[49] Alonso SR, Tracey L, Ortiz P, Perez-Gomez B, Palacios J, Pollan M (2007). A high-throughput study in melanoma identifies epithelial-mesenchymal transition as a major determinant of metastasis. Cancer Res..

[50] Panina Y, Germond A, Masui S, Watanabe TM (2018). Validation of common housekeeping genes as reference for qPCR gene expression analysis during iPS reprogramming process. Sci. Rep..

[51] Watanabe G, Saito Y, Madaule P, Ishizaki T, Fujisawa K, Morii N (1996). Protein kinase N (PKN) and PKN-related protein rhophilin as targets of small GTPase Rho. Science.

[52] Herraiz, C., Calvo, F., Pandya, P., Cantelli, G., Rodriguez-Hernandez, I., Orgaz, J. L. et al. Reactivation of p53 by a cytoskeletal sensor to control the balance between DNA damage and tumor dissemination. *J. Natl Cancer. Inst.* **108**, djv289 (2016). 10.1093/jnci/djv289 PMC4712681 26464464

[53] Dhomen N, Reis-Filho JS, da Rocha Dias S, Hayward R, Savage K, Delmas V (2009). Oncogenic Braf induces melanocyte senescence and melanoma in mice. Cancer Cell.

[54] Golombek SK, May JN, Theek B, Appold L, Drude N, Kiessling F (2018). Tumor targeting via EPR: strategies to enhance patient responses. Adv. Drug Deliv. Rev..

[55] Moriya H (2015). Quantitative nature of overexpression experiments. Mol. Biol. Cell.

[56] Graessl M, Koch J, Calderon A, Kamps D, Banerjee S, Mazel T (2017). An excitable Rho GTPase signaling network generates dynamic subcellular contraction patterns. J. Cell Biol..

[57] Routhier A, Astuccio M, Lahey D, Monfredo N, Johnson A, Callahan W (2010). Pharmacological inhibition of Rho-kinase signaling with Y-27632 blocks melanoma tumor growth. Oncol. Rep..

[58] Lavaud J, Henry M, Coll JL, Josserand V (2017). Exploration of melanoma metastases in mice brains using endogenous contrast photoacoustic imaging. Int J. Pharm..

[59] Iafrate, M. & Fruhwirth, G. O. How non-invasive in vivo cell tracking supports the development and translation of cancer immunotherapies. *Front. Physiol*. **11**, 154 (2020). 10.3389/fphys.2020.00154 PMC7152671 32327996

[60] Edmonds S, Volpe A, Shmeeda H, Parente-Pereira AC, Radia R, Baguna-Torres J (2016). Exploiting the metal-chelating properties of the drug cargo for in vivo positron emission tomography imaging of liposomal nanomedicines. ACS Nano.

[61] Man F, Lim L, Volpe A, Gabizon A, Shmeeda H, Draper B (2019). In vivo PET tracking of (89)Zr-Labeled Vgamma9Vdelta2 T cells to mouse xenograft breast tumors activated with liposomal alendronate. Mol. Ther..

[62] Thunemann M, Schorg BF, Feil S, Lin Y, Voelkl J, Golla M (2017). Cre/lox-assisted non-invasive in vivo tracking of specific cell populations by positron emission tomography. Nat. Commun..

[63] Ikenoya M, Hidaka H, Hosoya T, Suzuki M, Yamamoto N, Sasaki Y (2002). Inhibition of rho-kinase-induced myristoylated alanine-rich C kinase substrate (MARCKS) phosphorylation in human neuronal cells by H-1152, a novel and specific Rho-kinase inhibitor. J. Neurochem..

[64] Yap TA, Walton MI, Grimshaw KM, Te Poele RH, Eve PD, Valenti MR (2012). AT13148 is a novel, oral multi-AGC kinase inhibitor with potent pharmacodynamic and antitumor activity. Clin. Cancer Res.

[65] Ishizaki T, Uehata M, Tamechika I, Keel J, Nonomura K, Maekawa M (2000). Pharmacological properties of Y-27632, a specific inhibitor of rho-associated kinases. Mol. Pharm..

[66] Stavenger RA, Cui H, Dowdell SE, Franz RG, Gaitanopoulos DE, Goodman KB (2007). Discovery of aminofurazan-azabenzimidazoles as inhibitors of Rho-kinase with high kinase selectivity and antihypertensive activity. J. Med. Chem..

