# Supplementary material for: A preclinical pipeline to evaluate migrastatics as therapeutic agents in metastatic melanoma
Source: Br J Cancer. 2021 Jun 25;125(5):699–713. doi: 10.1038/s41416-021-01442-6 (PMC8405734; doi:10.1038/s41416-021-01442-6)
Supplement: Supplementary file 1 — Supplementary Materials [file 41416_2021_1442_MOESM1_ESM.pdf]

SUPPLEMENTARY MATERIALS

Contents

Supplementary Methods:

Additional methods used to perform the work in the manuscript. These methods have been reported before and are listed here to briefly report technical detail.

Supplementary Figures:

Supplementary Fig.S1. Correlation between gene expression and mutational status in melanoma patients.

Supplementary Fig.S2. Additional 4599.NC characterization experiments.

Supplementary Fig.S3. Effects of various ROCK inhibitors on A375M2 melanoma cell growth.

Supplementary Fig.S4. *In vivo* imaging enables quantification of the migrastatic effects of GSK269962.

Supplementary Fig.S5. Animal weights during drug treatment.

Supplementary Fig.S6. *Ex vivo* analysis of contractility in primary tumour tissues of animals treated with GSK269962.

Supplementary Tables S1, S2, S3, S4, S5. Tabulated data underlying graphical representations in Fig.1b including statistical information.

Supplementary Tab.S6. Additional data to the SILAC analyses including statistical information.

SUPPLEMENTARY METHODS

Flow Cytometry. Cells were lifted, washed twice in PBS and resuspended in PBS containing 2% (v/v) FBS and 0.1% (w/v) ethylenediaminetetraacetic acid (EDTA). Cells were analyzed on a FACSCanto flow cytometer (BD Bioscience) using appropriate channels settings for mCherry. Data was analysed using Flowing Software (http://flowingsoftware.btk.fi/).

Determination of NIS function by radiotracer uptake. NIS function was assessed by $^{99m}TcO_4^-$ uptake (50kBq/mL per 10^6 cells) as previously described [1]. Uptake specificity was tested after 30min preincubation with and in the presence of the competitive substrate sodium perchlorate (5µM). Radiotracer uptake was compared to a reference cell line, which was previously described (MTLn3E.Δ34; [1]).

Immunoblot analysis. Cells were lysed in 1.5-fold lithium dodecyl sulphate (LDS)-based sample buffer (92.5mM Tris/HCl pH 8.5, 0.75% (w/w) LDS, 3.75% (w/w) glycerol, 0.5mM

EDTA, 0.2mM sodium orthovanadate, 10nM calyculin A, 50mM NaF, 1040µM AEBSF, 800nM aprotinin, 40µM bestain, 14µM E64 protease inhibitor, 20µM leupeptin and 15µM pepstatin A) and lysates heated at 95°C for 20min. Lysate protein concentrations were determined using the BCA protein assay (Thermo Fisher, UK). Equal amounts of protein were supplemented with 100mM DTT and 60µM Serva Blue G250 (Serva, Germany), heated to 95°C for 5min, and proteins separated by SDS-PAGE before being transferred to PVDF Immobilon-P membranes (EMD Millipore, UK). Membranes were blocked with 4% (w/w) BSA in Tris-buffered saline containing 0.2% (w/v) Tween-20 (TBS-T) for 30min, incubated with indicated primary antibody overnight at 4°C, washed four times with TBS-T, and incubated with a corresponding secondary antibody conjugated to Alexa750 for 60min at room temperature. Signals were detected using an Odyssey Near-Infrared Fluorescence Imager (LI-COR, USA).

Microscopy of cells. For co-localization analysis with a plasma membrane marker, cells were grown on acid-treated glass coverslips and fixed in 4% (w/v) paraformaldehyde (PFA; 5min) before plasma membrane staining using wheat germ agglutinin (WGA) conjugated to AlexaFluor488 (Invitrogen, UK; 0.67µg/mL, 10min) and nuclei staining using Hoechst 33342 (Merck, Germany; 1.0µg/mL, 10min). Samples were rinsed twice with PBS and deionized water before mounting with Mowiol containing 2.5% (w/v) Dabco as an antifade. Samples were imaged on a Leica TCS SP5 confocal microscope equipped with a HCX Plan Apo 63x/1.4NA oil immersion objective (Leica, Germany) and appropriate filter sets for Hoechst 33342/DAPI, AlexaFluor488/GFP and mCherry/TexasRed. Morphologiuc analysis of cells on collagen was performed as previously described [2].

Cell survival in the presence of ROCK inhibitors. $3 \cdot 10^4$ 4599.NC cells were seeded into the wells of 12-well plates the day before the assay, and then treated with the indicated ROCK inhibitors or vehicle only for 96h in the presence of fully supplemented growth medium under normal cell culture atmosphere. Subsequently, cells were fixed with 3.7% (w/v) formaldehyde followed by staining with 0.25% (w/v) crystal violet. Cells were dried over night before solubilisation in 10% (v/v) acetic acid and absorbance measurements (595nm) of the resultant solutions.

Analysis of radioactivity from harvested tissues. For terminal *ex vivo* γ-counting, animals were euthanized 90min following radiotracer administration. All harvested tissues were weighed, immediately immersed in 3.7% (w/v) formaldehyde solution, γ-counted (1282 Compugamma, LKB, Australia) alongside radioactivity calibration standards, and percent injected dose per gram (%ID/g) was calculated for each tissue.

References in the Supplement

1 Fruhwirth, G. O., Diocou, S., Blower, P. J., Ng, T. & Mullen, G. E. A whole-body dual-modality radionuclide optical strategy for preclinical imaging of metastasis and heterogeneous treatment response in different microenvironments. *J Nucl Med* **55**, 686-694 (2014).

2 Georgouli, M., Herraiz, C., Crosas-Molist, E., Fanshawe, B., Maiques, O., Perdrix, A. *et al.* Regional Activation of Myosin II in Cancer Cells Drives Tumor Progression via a Secretory Cross-Talk with the Immune Microenvironment. *Cell* **176**, 757-774 e723 (2019).

3 Tyanova, S., Temu, T. & Cox, J. The MaxQuant computational platform for mass spectrometry-based shotgun proteomics. *Nat Protoc* **11**, 2301-2319 (2016).

4 Tyanova, S., Temu, T., Sinitcyn, P., Carlson, A., Hein, M. Y., Geiger, T. *et al.* The Perseus computational platform for comprehensive analysis of (prote)omics data. *Nat Methods* **13**, 731-740 (2016).

SUPPLEMENTARY FIGURE LEGENDS

Supplementary Fig.S1. Correlation between gene expression and mutational status in melanoma patients. mRNA expression levels of ARHGEF1 (a), ARHGEF2 (b), ARHGEF11 (c), ROCK1 (d) and ROCK2 (e) according to the mutational status of BRAF and NRAS in melanoma patients from TCGA. Graphs show mean and SEM (each dot represents a patient). *P*-values were calculated using one-way ANOVA. For all panels *p*-values are <0.05 (*), <0.01 (**) and non-significant (ns) as indicated on relevant comparisons.

Supplementary Fig.S2. Additional 4599.NC characterization experiments | (a) Flow cytometric analysis of sorted 4599.NC cells (black line) *versus* parental 4599 cells (shaded gray) one week after preparative FACS sorting (top panel) and four weeks after preparative FACS sorting (bottom panel). **(b)** *In vivo* growth comparison of tumours established from either parental 4599 (black) or reporter gene-expressing 4599.NC (red) cells. Shown are tumour growth curves of tumours established in male NSG mice; *N*=4 per cohort, error bars are SD. **(c)** *Ex vivo* γ-counting of harvested tissues from the animals in Fig.4; shown are cumulative data (*N*=4) with error bars representing SEM.

Supplementary Fig.S3. Effects of various ROCK inhibitors on A375M2 melanoma cell growth | Proliferation assays of human A375M2 melanoma cells in the presence of different concentrations of the ROCK inhibitors **(a)** AT13148 and **(b)** GSK269962. Error bars are SD; N=3. EC_{50} including 95% confidence intervals [lower;higher end of range] were calculated using the variable slope dose-response model built into Graphpad Prism software v7.01.

Supplementary Fig.S4. *In vivo* imaging enables quantification of the migrastatic effects of GSK269962 | (a) Diagram of the experimental design with green arrows indicating intraperitoneal ROCK inhibitor GSK269962 administration (every other day at 44µmol/kg and black arrows indicate *in vivo* imaging time points. **(b)** Tumour volume on day 10 measured using callipers. Data shown are mean tumour sizes of cohorts after animal randomization. Red represents the future vehicle cohort while green represent the future treatment cohort. Treatments started on the next day after *in vivo* SPECT/CT imaging to obtain baseline data. **(c)** Live tumour volumes (LTV) and **(d)** radiotracer uptake in tumours at day 10 as determined using SPECT/CT imaging. Colour code as in (b). Error bars represent SEM (*N*=4). **(e)** Representative maximum intensity projection overlay images of $^{99m}TcO_4^-$-afforded NIS-SPECT and CT before treatment start (day 10 'baseline'/left) and at the end of the experiment (day 29/right). Right animals in both panels are GSK269962-treated while left animals represent control animals. Solid yellow arrows indicated tumours (T) while dotted yellow

arrows indicate metastases in lung and axillary lymph nodes (LN)). Organs labelled in white indicate endogenous NIS-expressing organs which did not interfere with sites of metastasis (thyroid and salivary glands (T/S), lachrymal glands (L), stomach (S), low levels in intestine (I) and testes (Te)). **(f)** 3D volume rendering of live tumour cells based on Otsu-thresholded SPECT signals from (e). Vehicle (top) and inhibitor-treated (bottom) boxes contain pseudo-coloured 3D-rendered volumes with tumours visualized in turquoise, lung metastases in gray, and lymph node metastases in red. **(g)** Live tumour volume (LTV) and **(h)** radiotracer uptake of tumours was quantified across all animals per cohort. Red represents vehicle- and green ROCK inhibitor-treated animals Error bars are SEM and *P*-values were calculated using two-tailed unpaired *t*-tests with Welch's correction; *N*=4 per cohort. **(i)** LTV and **(j)** radiotracer uptake in all detected lung metastases combined. Colour code and statistics as in (g/h).

Supplementary Fig.S5. Animal weights during drug treatments | Time course of average animal weights of all animals in the corresponding studies. Treatments were with either **(a)** Y27632 corresponding to Fig.5 (*N*=4 per cohort) or **(b)** GSK269962 corresponding to Fig.S3 (*N*=4 per cohort). Averages are with error bars indicating SD.

Supplementary Fig.S6. *Ex vivo* analysis of contractility in primary tumour tissues of animals treated with GSK269962. | Histology Score (H-score) for phospho-MLC-stained tumour sections covering TB, IF and DIF; a representative stained image is shown (top) as well as a corresponding score map (bottom) and magnified insets for each region (middle column). Scale bars are 250µm and 50µm for the insets. The right column shows data of the corresponding cumulative analyses for each region. *P*-values were calculated using unpaired *t*-tests and two-way ANOVA with Sidak's multiple comparison where applicable. For all panels *P*-values are <0.05 (*), <0.01 (**), <0.001 (***), or <0.0001 (****) as indicated on relevant comparisons.

Fig.S1

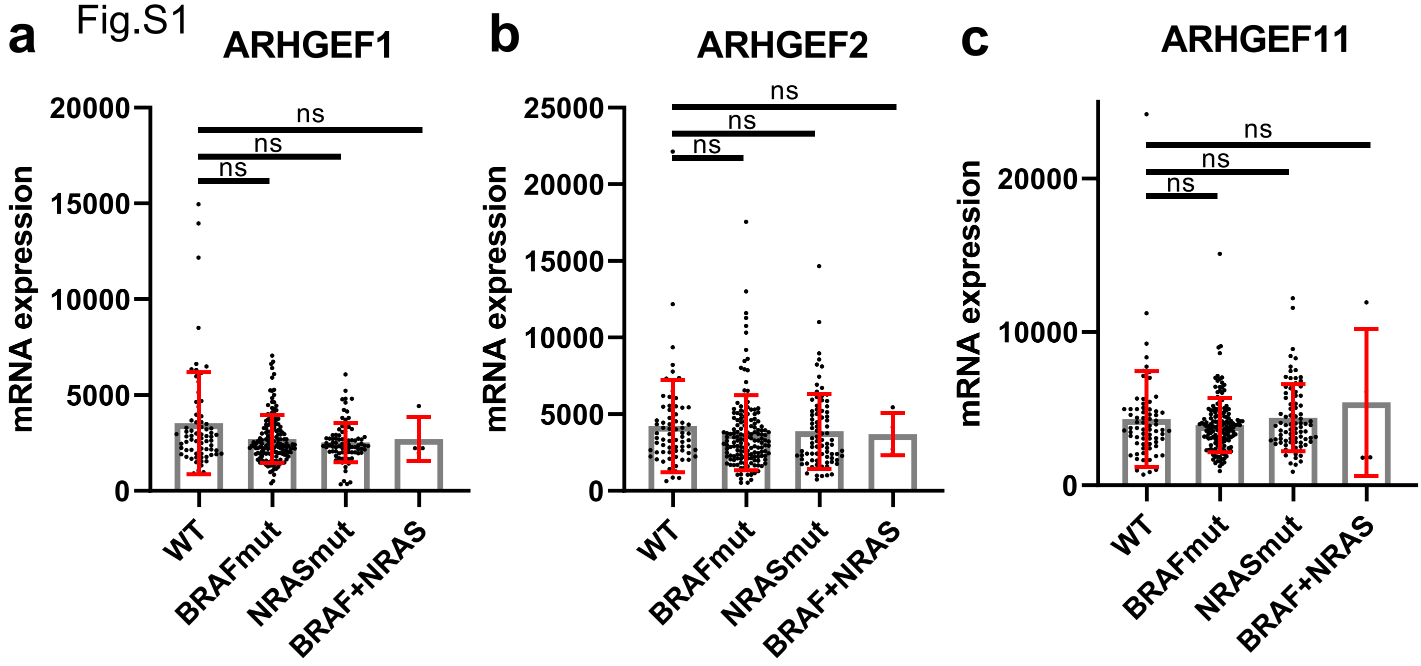

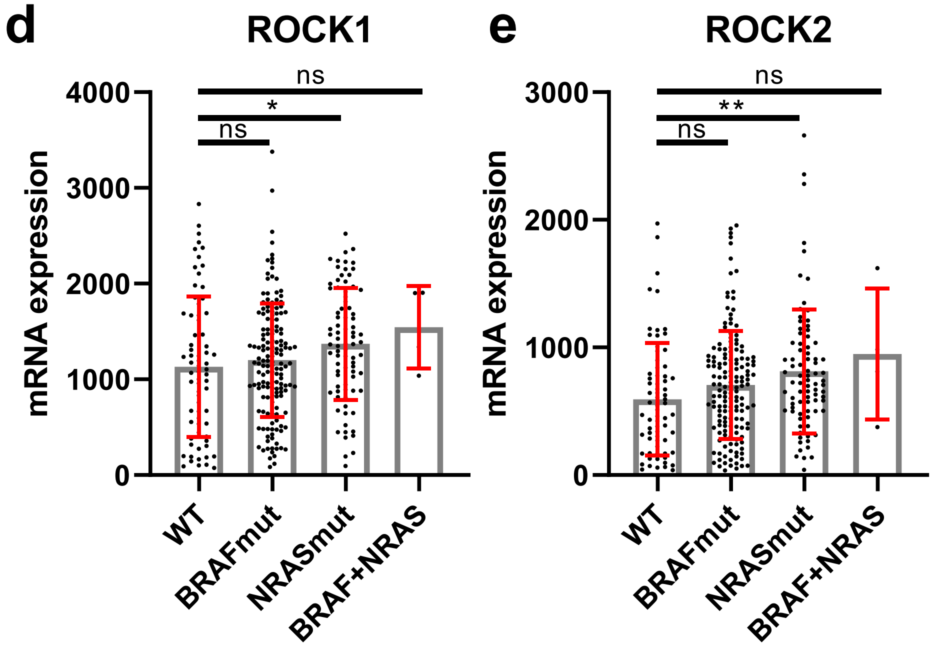

Patients analysed (TCGA)	
Total	311
Wildtype	70
BRAF mutant	154
NRAS mutant	82
BRAF/NRAS mut.	4

All patients treatment naïve and samples >70% tumour cells

Fig.S2

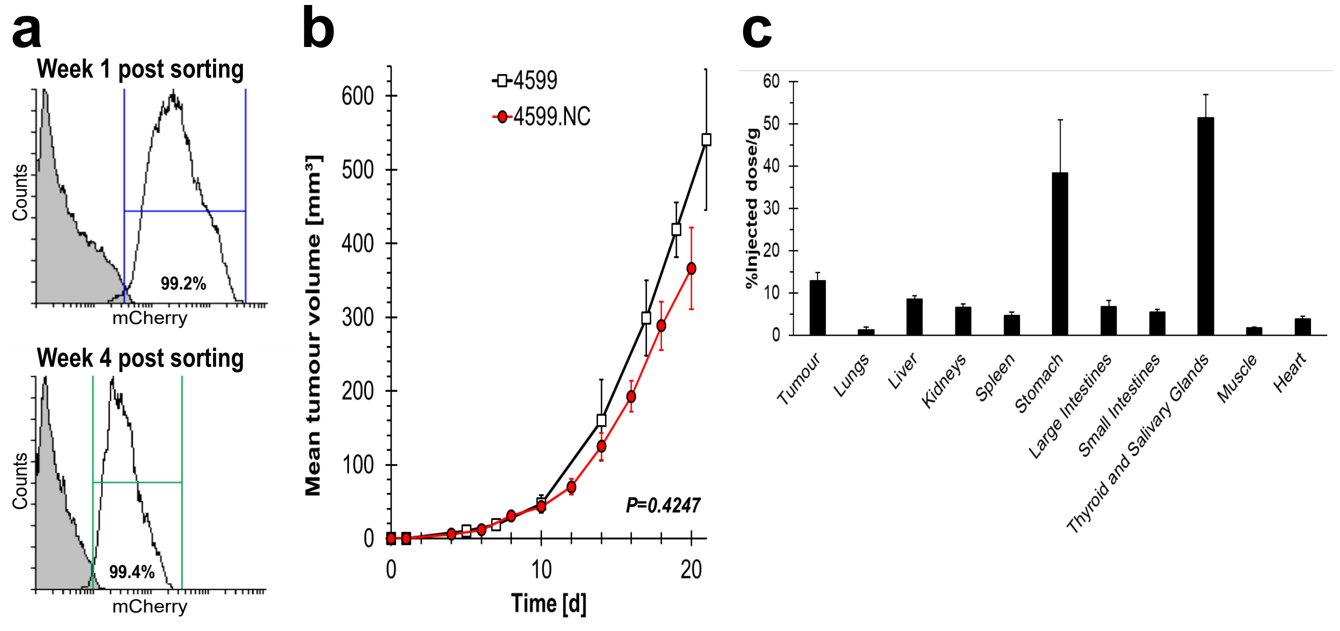
a
Week 1 post sorting
Counts
99.2%
mCherry
Week 4 post sorting
Counts
99.4%
mCherry
b
4599
4599.NC
Mean tumour volume [mm³]
600
500
400
300
200
100
0
P=0.4247
0
10
20
Time [d]
c
%Injected dose/g
60
50
40
30
20
10
0
Tumour
Lungs
Liver
Kidneys
Spleen
Stomach
Large Intestines
Small Intestines
Thyroid and Salivary Glands
Muscle
Heart

Fig.S3

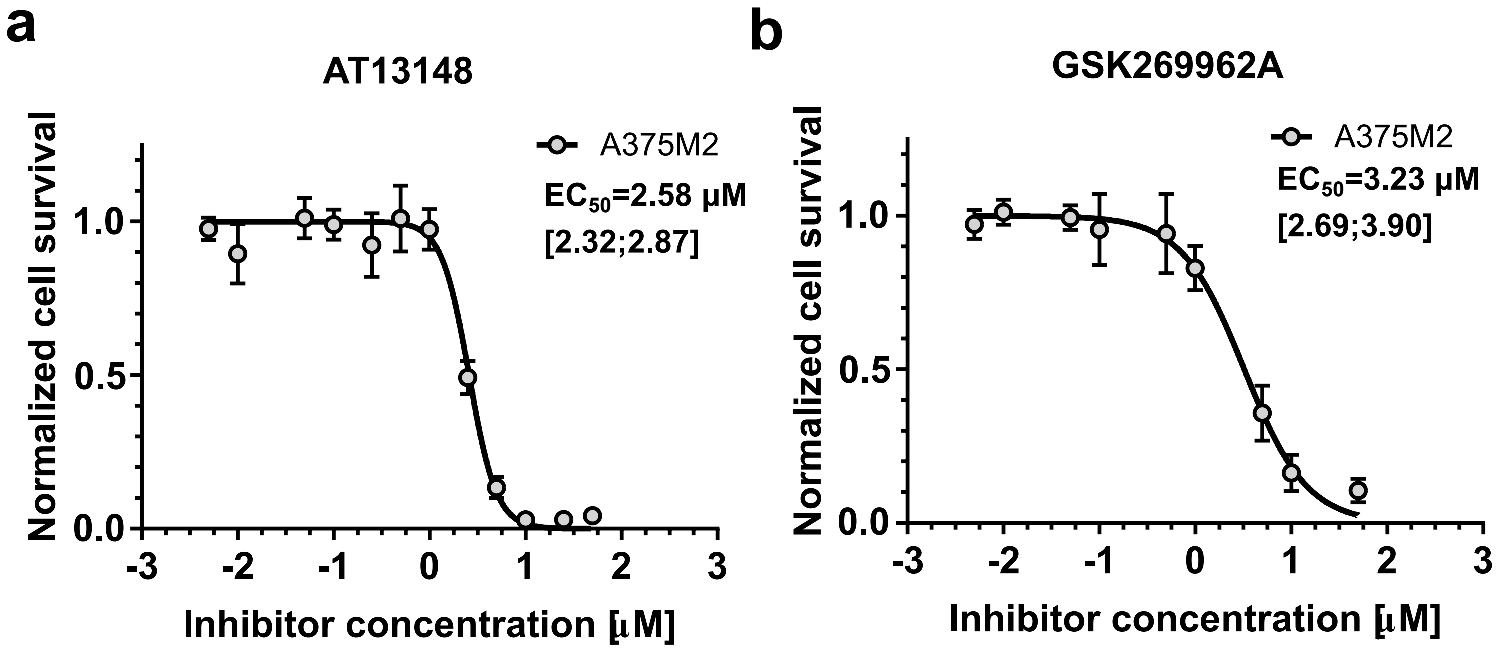
a
AT13148
A375M2
EC50=2.58 µM
[2.32;2.87]
Normalized cell survival
Inhibitor concentration [µM]
b
GSK269962A
A375M2
EC50=3.23 µM
[2.69;3.90]
Normalized cell survival
Inhibitor concentration [µM]

Fig.S4

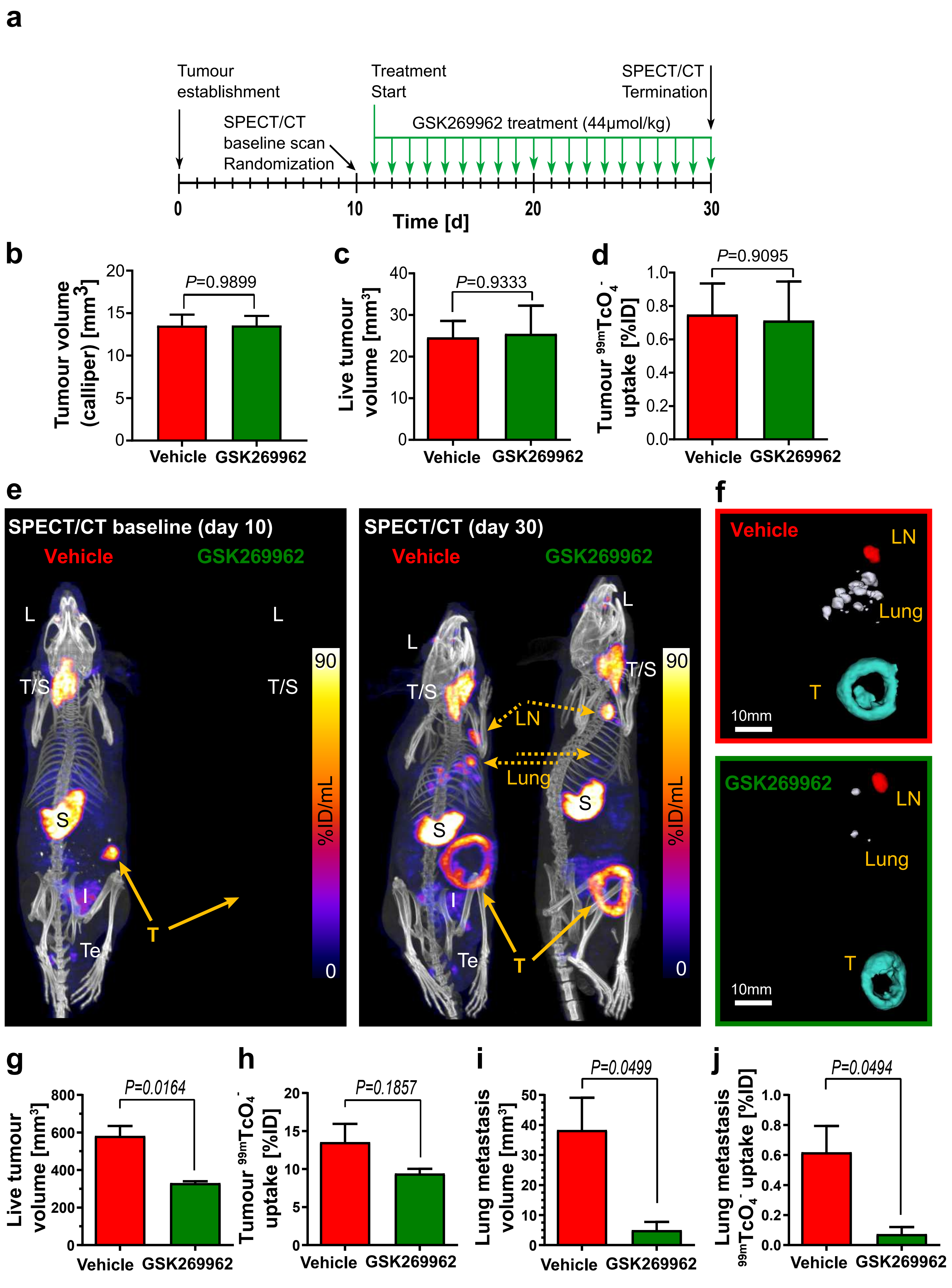
a
Tumour establishment
Treatment Start
SPECT/CT Termination
SPECT/CT baseline scan Randomization
GSK269962 treatment (44μmol/kg)
0
10
20
30
Time [d]
b
Tumour volume (calliper) [mm3]
P=0.9899
Vehicle
GSK269962
c
Live tumour volume [mm3]
P=0.9333
d
Tumour 99mTcO4- uptake [%ID]
P=0.9095
e
SPECT/CT baseline (day 10)
SPECT/CT (day 30)
Vehicle
GSK269962
L
T/S
S
I
Te
T
LN
Lung
90
0
%ID/mL
f
Vehicle
GSK269962
LN
Lung
T
10mm
g
Live tumour volume [mm3]
P=0.0164
h
Tumour 99mTcO4- uptake [%ID]
P=0.1857
i
Lung metastasis volume [mm3]
P=0.0499
j
Lung metastasis 99mTcO4- uptake [%ID]
P=0.0494

Fig.S5

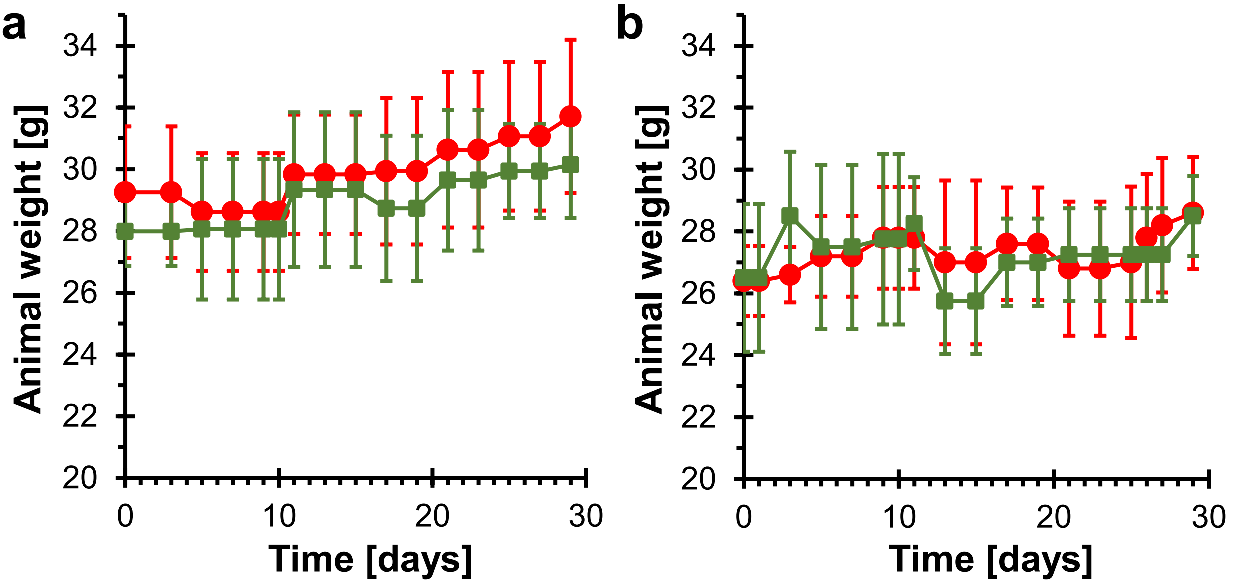

Fig.S6

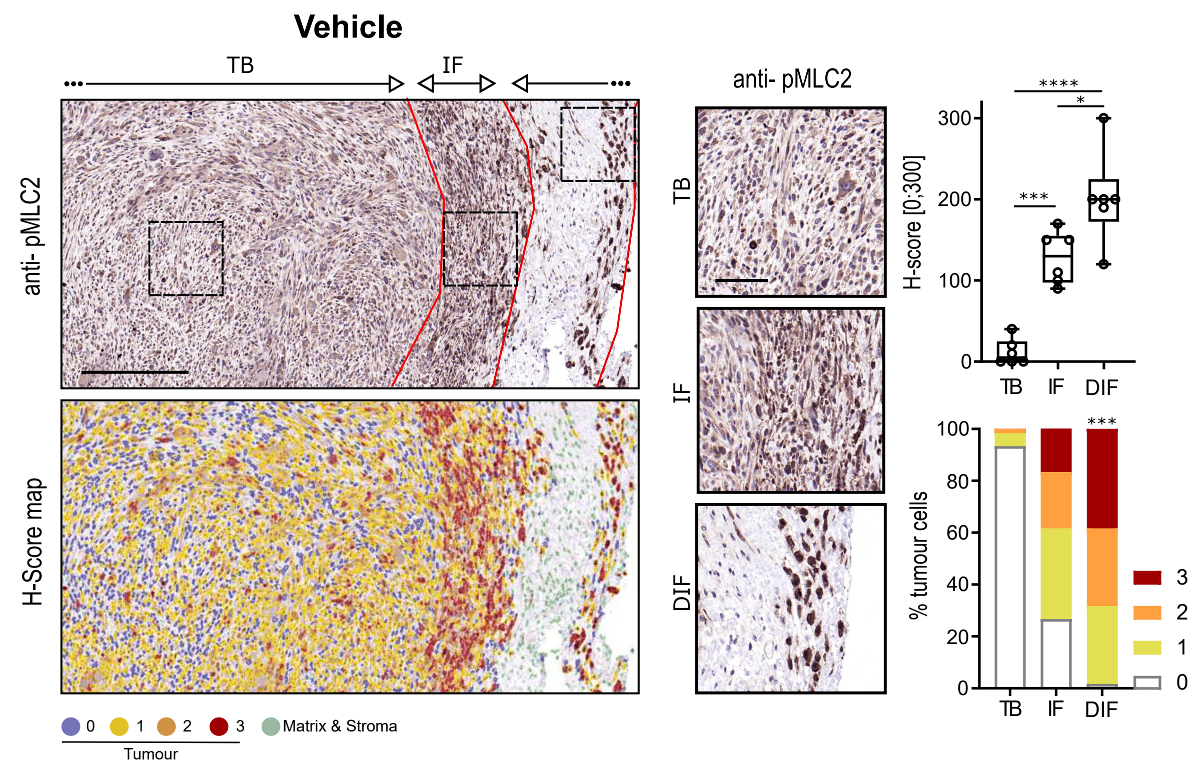
Vehicle
TB
IF
anti- pMLC2
H-Score map
0
1
2
3
Matrix & Stroma
Tumour
anti- pMLC2
TB
IF
DIF
H-score [0;300]
300
200
100
0
****
*
***
TB
IF
DIF
% tumour cells
100
80
60
40
20
0
***
3
2
1
0
TB
IF
DIF

Supplementary table S1. Tabulated data used for the generation of the figure panel reporting on RhoGTPase gene expression in Fig.1b | Fold change for the indicated comparison in the also indicated database is given together with associated p-values. A difference is considered significant if *p* <0.05. If *p* -values are tabulated as "0.000" then they were <0.001.

		NEVUS vs. NORMAL				PRIMARY vs. NORMAL						PRIMARY vs. NEVUS				MET vs. PRIMARY								MELANOMA vs. MELANOCYTE			
		Talantov		Kabbarah		Kabbarah		Talantov		Riker		Talantov		Kabbarah		Riker		Kabbarah		Xu		TCGA		Philadelphia		Mannheim	
	Gene_Symbol	Fold change	*p* -value	Fold change	*p* -value	Fold change	*p* -value	Fold change	*p* -value	Fold change	*p* -value	Fold change	*p* -value	Fold change	*p* -value	Fold change	*p* -value	Fold change	*p* -value	Fold change	*p* -value	Fold change	*p* -value	Fold change	*p* -value	Fold change	*p* -value
RHO GEFs	ABR	1.301	0.101	1.224	0.185	1.342	0.050	1.574	0.000	0.975	0.929	1.210	0.030	1.097	0.488	1.017	0.910	0.697	0.001	0.817	0.076	0.796	0.001	0.671	0.040	0.766	0.131
	AKAP13	1.260	0.430	0.946	0.748	1.033	0.848	1.271	0.249	1.397	0.353	1.009	0.953	1.092	0.572	1.035	0.867	0.821	0.017	0.788	0.197	1.320	0.000	0.908	0.580	0.818	0.093
	ARHGEF1	1.264	0.060	1.268	0.079	1.581	0.000	1.713	0.000	1.131	0.555	1.355	0.000	1.247	0.003	1.109	0.427	0.954	0.460	0.940	0.355	0.946	0.351	0.773	0.007	1.035	0.687
	ARHGEF10	0.516	0.001	0.765	0.039	0.605	0.000	0.525	0.023	0.903	0.695	1.017	0.925	0.790	0.029	1.068	0.669	1.147	0.100	1.445	0.026	1.117	0.177	1.538	0.003	2.045	0.000
	ARHGEF11	1.952	0.008	1.201	0.133	1.399	0.015	2.430	0.000	1.364	0.381	1.245	0.056	1.164	0.197	0.993	0.971	1.118	0.185	1.200	0.063	0.889	0.073	1.331	0.065	1.487	0.014
	ARHGEF12	1.665	0.000	1.152	0.392	1.320	0.005	1.347	0.070	0.773	0.252	0.809	0.048	1.145	0.109	0.841	0.218	0.832	0.001	0.791	0.015	1.021	0.752	1.054	0.693	1.229	0.046
	ARHGEF15	0.641	0.005	1.117	0.501	0.999	0.994	0.611	0.000	1.299	0.383	0.954	0.562	0.894	0.391	0.672	0.007	0.922	0.274	0.993	0.948	0.946	0.604	1.070	0.570	1.780	0.000
	ARHGEF16	1.557	0.010	1.027	0.810	1.056	0.635	1.039	0.775	0.604	0.219	0.667	0.000	1.028	0.792	0.813	0.392	0.988	0.861	0.739	0.021	0.536	0.003	0.620	0.000	1.339	0.000
	ARHGEF17	0.864	0.663	0.831	0.392	0.791	0.257	1.683	0.001	1.553	0.282	1.948	0.000	0.952	0.799	0.948	0.812	0.626	0.000	0.890	0.409	1.050	0.556	0.388	0.000	1.623	0.000
	ARHGEF26	3.339	0.010	0.609	0.204	0.625	0.068	1.008	0.980	0.455	0.065	0.302	0.000	1.026	0.892	0.844	0.517	1.212	0.146	1.311	0.215	1.007	0.974	1.746	0.000	1.687	0.000
	ARHGEF3	0.381	0.003	1.500	0.007	1.538	0.003	0.240	0.000	0.934	0.848	0.630	0.004	1.026	0.841	0.922	0.655	0.839	0.072	1.016	0.923	1.526	0.000	2.032	0.007	7.115	0.000
	ARHGEF5	1.335	0.476	0.380	0.000	0.290	0.000	0.170	0.000	0.192	0.004	0.127	0.000	0.761	0.061	0.617	0.132	0.699	0.005	0.626	0.004	0.877	0.576	0.945	0.760	2.694	0.007
	PLEKHG6	0.842	0.397	0.851	0.131	0.661	0.003	0.648	0.001	0.605	0.082	0.770	0.017	0.777	0.039	0.786	0.056	0.916	0.214	0.848	0.111	0.418	0.000	1.059	0.417	1.071	0.143
	OBSCN	0.726	0.202	0.942	0.725	1.247	0.111	0.810	0.316	0.749	0.462	1.116	0.311	1.324	0.027	0.946	0.771	0.750	0.000	0.987	0.889	1.087	0.552	0.123	0.000	2.002	0.000
	NET1	0.703	0.019	0.976	0.873	0.538	0.001	0.189	0.000	0.429	0.018	0.269	0.000	0.551	0.000	0.604	0.002	0.464	0.000	0.553	0.000	1.102	0.259	1.354	0.207	2.590	0.002
	RGNEF	0.703	0.290	1.029	0.899	0.946	0.743	0.372	0.000	0.571	0.095	0.529	0.001	0.920	0.622	0.466	0.000	1.061	0.638	0.760	0.103	#VALUE!	NA	0.925	0.622	1.854	0.000
	VAV3	0.727	0.213	0.690	0.009	0.489	0.000	0.148	0.000	0.172	0.001	0.203	0.000	0.709	0.010	0.696	0.153	0.879	0.100	0.929	0.545	1.454	0.031	1.654	0.064	3.886	0.009
RAC GEFs	ARHGEF6	0.593	0.002	1.238	0.035	1.272	0.006	0.466	0.000	0.997	0.994	0.786	0.008	1.027	0.725	0.947	0.819	0.911	0.242	0.882	0.320	1.815	0.000	0.782	0.047	1.363	0.228
	ARHGEF7	1.019	0.852	0.947	0.767	1.165	0.225	0.884	0.296	1.136	0.536	0.867	0.051	1.230	0.101	1.095	0.410	1.239	0.002	1.265	0.000	0.946	0.247	1.667	0.000	1.975	0.000
	DOCK1	0.942	0.673	0.870	0.187	0.627	0.000	0.774	0.019	0.609	0.016	0.822	0.004	0.721	0.000	1.201	0.160	0.989	0.852	1.080	0.253	1.301	0.000	0.986	0.879	1.006	0.936
	DOCK2	0.669	0.396	0.771	0.327	1.330	0.305	0.368	0.044	2.033	0.200	0.549	0.073	1.726	0.043	0.981	0.954	0.823	0.324	1.213	0.518	2.676	0.000	0.495	0.001	3.467	0.106
	DOCK3	0.952	0.766	0.602	0.044	0.769	0.195	0.858	0.238	1.012	0.977	0.901	0.274	1.278	0.192	0.586	0.024	1.073	0.584	0.831	0.110	1.094	0.526	0.452	0.000	3.167	0.000
	DOCK4	0.413	0.000	1.026	0.832	1.062	0.614	0.830	0.247	1.806	0.184	2.010	0.000	1.035	0.745	1.506	0.097	1.549	0.000	1.785	0.000	1.705	0.000	2.732	0.000	3.617	0.000
	DOCK5	0.418	0.039	1.203	0.246	1.306	0.046	0.792	0.351	1.829	0.023	1.893	0.002	1.086	0.516	1.061	0.661	1.038	0.636	1.010	0.940	1.117	0.288	0.834	0.127	1.290	0.007
	FARP1	0.985	0.956	1.470	0.041	1.748	0.008	3.841	0.000	2.790	0.001	3.899	0.000	1.189	0.331	0.964	0.793	0.918	0.597	0.944	0.740	0.973	0.749	0.692	0.075	0.927	0.670
	FARP2	1.361	0.060	1.344	0.012	1.347	0.004	2.142	0.000	1.154	0.672	1.574	0.000	1.003	0.977	0.953	0.799	0.941	0.471	0.921	0.515	0.920	0.363	0.588	0.044	1.030	0.905
	ITSN2	0.803	0.231	1.073	0.658	1.021	0.905	0.612	0.018	0.851	0.696	0.762	0.042	0.951	0.751	0.997	0.989	1.267	0.003	1.332	0.056	1.434	0.000	0.945	0.552	1.924	0.000
	RASGRF1	0.635	0.007	1.829	0.031	2.257	0.002	1.130	0.384	1.235	0.482	1.782	0.000	1.234	0.317	1.062	0.697	0.933	0.491	1.321	0.015	1.414	0.109	0.683	0.000	1.061	0.523
	SOS1	0.538	0.000	0.846	0.189	1.038	0.747	0.660	0.000	0.867	0.633	1.228	0.002	1.228	0.066	0.999	0.996	1.206	0.038	1.363	0.001	1.474	0.000	1.822	0.003	2.785	0.000
	SWAP70	0.827	0.425	1.769	0.012	1.289	0.039	0.495	0.009	1.524	0.133	0.598	0.004	0.729	0.017	0.831	0.479	1.288	0.026	1.104	0.539	1.214	0.027	0.880	0.387	0.768	0.007
	TIAM2	0.664	0.034	1.413	0.038	1.383	0.029	0.672	0.001	1.276	0.559	1.013	0.881	0.978	0.862	1.015	0.928	1.108	0.101	1.323	0.002	1.569	0.003	1.512	0.000	1.065	0.586
CDC42 GEFs	ARHGEF9	1.002	0.994	1.227	0.273	0.869	0.342	0.748	0.091	0.330	0.003	0.746	0.059	0.708	0.020	1.128	0.616	1.098	0.417	0.619	0.002	0.966	0.646	0.766	0.077	1.372	0.000
	DNMBP	0.695	0.008	0.615	0.001	0.539	0.000	0.380	0.000	0.522	0.012	0.547	0.000	0.875	0.096	0.822	0.118	0.914	0.090	1.097	0.350	1.035	0.547	0.902	0.538	1.064	0.713
	DOCK9	0.395	0.000	0.523	0.000	0.439	0.000	0.154	0.000	0.404	0.052	0.389	0.000	0.838	0.186	0.612	0.045	0.734	0.000	0.720	0.003	1.050	0.564	1.525	0.059	3.950	0.000
	FGD1	1.080	0.558	1.130	0.268	1.248	0.016	2.114	0.000	1.580	0.151	1.957	0.000	1.104	0.229	0.919	0.571	1.081	0.167	1.225	0.041	0.841	0.011	0.821	0.199	1.388	0.017
	FGD2	0.548	0.002	0.982	0.903	1.073	0.554	0.522	0.001	1.641	0.326	0.952	0.715	1.093	0.425	1.004	0.987	1.141	0.068	1.135	0.278	1.919	0.000	1.483	0.001	1.441	0.000
	FGD6	0.962	0.848	0.762	0.147	0.589	0.002	0.445	0.004	0.511	0.059	0.462	0.000	0.773	0.090	0.615	0.002	0.977	0.766	1.033	0.869	1.376	0.001	1.944	0.078	2.229	0.000
	ARHGEF40	1.520	0.036	1.745	0.000	1.576	0.000	2.073	0.000	2.996	0.029	1.364	0.000	0.904	0.384	1.108	0.706	1.022	0.814	1.106	0.391	0.914	0.259	0.698	0.001	0.898	0.325
	ARHGEF38	1.091	0.666	1.331	0.077	1.365	0.034	1.137	0.511	0.495	0.167	1.042	0.766	1.026	0.831	1.368	0.193	1.133	0.066	1.174	0.213	1.028	0.348	1.401	0.007	1.180	0.087
	KALRN	0.807	0.572	1.689	0.112	1.877	0.006	0.960	0.890	0.965	0.930	1.190	0.415	1.111	0.589	0.969	0.898	1.459	0.000	1.594	0.026	1.338	0.033	0.915	0.498	2.282	0.000
	ITSN1	0.649	0.116	0.534	0.017	0.450	0.000	0.458	0.006	0.547	0.232	0.706	0.078	0.843	0.048	0.967	0.866	1.039	0.598	0.949	0.734	1.125	0.043	1.026	0.835	2.104	0.000
	MCF2L2	0.774	0.661	0.692	0.148	0.941	0.746	1.084	0.898	0.969	0.953	1.401	0.439	1.360	0.098	1.007	0.979	0.826	0.098	0.814	0.448	1.117	0.457	0.437	0.000	0.924	0.357
	SOS2	0.965	0.739	1.092	0.338	0.878	0.122	0.866	0.100	0.679	0.213	0.897	0.071	0.804	0.006	1.135	0.283	1.103	0.051	1.167	0.101	1.181	0.023	0.654	0.134	1.568	0.025
RHO/ RAC GEFs	ARHGEF18	1.033	0.762	0.892	0.319	0.997	0.973	1.498	0.000	0.904	0.641	1.449	0.000	1.118	0.206	1.207	0.128	0.930	0.381	1.238	0.008	1.050	0.345	0.951	0.735	1.669	0.000
	ARHGEF2	0.787	0.198	1.210	0.044	1.555	0.000	1.709	0.000	2.486	0.020	2.171	0.000	1.285	0.006	1.427	0.027	1.243	0.000	1.464	0.000	1.338	0.000	1.017	0.868	1.664	0.000
	TRIO	0.564	0.003	0.764	0.057	0.730	0.005	0.825	0.159	1.660	0.058	1.463	0.000	0.955	0.632	0.833	0.226	1.352	0.001	1.238	0.027	1.231	0.010	0.546	0.003	0.824	0.223
RHO/ CDC42 GEFs	MCF2L	0.640	0.001	0.834	0.104	0.878	0.326	0.760	0.040	0.867	0.703	1.187	0.056	1.052	0.674	0.851	0.521	0.783	0.041	0.898	0.376	0.536	0.001	0.444	0.022	0.492	0.031
RHO/RAC1/ CDC42 GEFs	ARHGEF4	0.818	0.391	0.518	0.001	0.335	0.000	0.132	0.000	0.137	0.003	0.161	0.000	0.647	0.062	0.465	0.019	0.299	0.000	0.298	0.000	0.283	0.000	1.116	0.445	1.238	0.087
	DEF6	1.635	0.172	0.734	0.020	0.852	0.221	0.659	0.159	0.383	0.047	0.403	0.000	1.160	0.212	0.977	0.928	0.672	0.000	0.549	0.001	1.257	0.094	0.524	0.000	2.137	0.000
	ECT2	0.544	0.008	0.840	0.150	1.189	0.286	0.628	0.165	1.510	0.365	1.155	0.502	1.415	0.022	1.684	0.019	1.777	0.000	2.083	0.000	1.454	0.002	2.457	0.006	7.126	0.000
	MCF2	0.677	0.296	1.195	0.325	1.086	0.561	0.805	0.509	1.292	0.678	1.189	0.431	0.909	0.475	1.053	0.865	1.461	0.000	1.275	0.243	1.149	0.526	0.494	0.018	1.115	0.574
	VAV2	2.218	0.017	1.863	0.021	1.767	0.003	1.331	0.384	0.673	0.446	0.600	0.014	0.948	0.742	1.581	0.166	1.003	0.981	0.832	0.290	1.173	0.136	0.610	0.000	2.119	0.000
	BCR	0.991	0.938	0.829	0.111	0.827	0.112	0.602	0.000	0.663	0.164	0.607	0.000	0.998	0.986	0.728	0.056	0.688	0.000	0.936	0.443	0.920	0.302	1.705	0.000	2.074	0.000
RAC/ CDC42 GEFs	DOCK10	8.312	0.000	3.704	0.000	3.241	0.000	7.375	0.000	1.753	0.156	0.887	0.543	0.875	0.499	1.708	0.004	1.156	0.414	1.271	0.279	1.485	0.002	2.242	0.001	2.957	0.000
	DOCK6	0.686	0.008	0.785	0.091	0.901	0.273	0.661	0.000	0.661	0.092	0.964	0.616	1.149	0.160	0.846	0.275	0.776	0.000	0.780	0.004	0.953	0.523	0.598	0.000	1.324	0.000
	PLEKHG3	0.896	0.615	1.255	0.296	1.237	0.261	1.063	0.696	0.962	0.909	1.187	0.093	0.986	0.929	0.688	0.160	1.163	0.086	0.898	0.512	0.732	0.002	0.423	0.000	1.054	0.320
	TIAM1	0.881	0.473	0.475	0.000	0.395	0.000	0.133	0.000	0.387	0.013	0.151	0.000	0.833	0.328	0.459	0.008	0.550	0.000	0.595	0.001	0.847	0.225	1.817	0.003	2.013	0.001
	VAV1	1.030	0.857	0.972	0.856	1.273	0.035	1.121	0.524	2.803	0.103	1.089	0.494	1.311	0.010	1.311	0.345	1.072	0.395	1.166	0.219	2.412	0.000	0.828	0.043	1.613	0.012

Supplementary table S2. Tabulated data used for the generation of the figure panel reporting on RhoGTPase gene expression in Fig.1b | Fold change for the indicated comparison in the also indicated database is given together with associated p-values. A difference is considered significant if *p* <0.05. If *p* -values are tabulated as "0.000" then they were <0.001.

		NEVUS vs. NORMAL				PRIMARY vs. NORMAL						PRIMARY vs. NEVUS				MET vs. PRIMARY								MELANOMA vs. MELANOCYTE			
		Talantov		Kabbarah		Kabbarah		Talantov		Riker		Talantov		Kabbarah		Riker		Kabbarah		Xu		TCGA		Philadelphia		Mannheim	
	Gene_Symbol	Fold change	*p* -value	Fold change	*p* -value	Fold change	*p* -value	Fold change	*p* -value	Fold change	*p* -value	Fold change	*p* -value	Fold change	*p* -value	Fold change	*p* -value	Fold change	*p* -value	Fold change	*p* -value	Fold change	*p* -value	Fold change	*p* -value	Fold change	*p* -value
RhoGTPases	RHOA	1.669	0.027	1.089	0.452	1.029	0.837	1.478	0.078	1.125	0.541	0.886	0.420	0.945	0.629	0.952	0.563	0.962	0.687	1.120	0.109	0.901	0.002	0.992	0.963	1.833	0.000
	RHOB	0.611	0.028	0.990	0.951	0.945	0.739	0.784	0.057	0.433	0.008	1.283	0.020	0.955	0.756	1.088	0.575	0.736	0.016	0.860	0.175	0.824	0.008	0.714	0.044	0.850	0.251
	RHOC	4.239	0.002	0.955	0.673	0.940	0.584	9.899	0.000	0.981	0.967	2.335	0.000	0.984	0.875	1.094	0.792	0.897	0.359	1.029	0.810	0.835	0.008	1.091	0.638	2.243	0.000
	RAC1	0.910	0.424	0.955	0.599	1.049	0.571	0.878	0.037	0.980	0.909	0.965	0.489	1.099	0.197	1.009	0.917	0.942	0.423	1.074	0.266	0.871	0.000	1.008	0.932	1.397	0.000
	RAC2	0.911	0.751	1.061	0.794	1.609	0.102	0.700	0.219	2.557	0.066	0.769	0.212	1.517	0.124	1.242	0.457	0.867	0.436	0.949	0.823	1.972	0.000	4.073	0.027	5.542	0.001
	RAC3	2.801	0.009	1.588	0.013	1.713	0.005	5.058	0.000	0.441	0.083	1.806	0.009	1.079	0.643	1.582	0.217	0.907	0.356	0.959	0.839	0.737	0.024	0.591	0.013	1.768	0.000
	CDC42	1.003	0.990	1.437	0.024	1.292	0.062	0.945	0.717	1.471	0.154	0.942	0.601	0.899	0.358	0.759	0.025	1.164	0.032	1.303	0.078	0.993	0.936	1.572	0.000	1.439	0.001

Supplementary table S3. Tabulated data used for the generation of the figure panel reporting on effectors in Fig.1b | Fold change for the indicated comparison in the also indicated database is given together with associated p-values. A difference is considered significant if *p* <0.05. If *p* -values are tabulated as "0.000" then they were <0.001.

		NEVUS vs. NORMAL				PRIMARY vs. NORMAL						PRIMARY vs. NEVUS				MET vs. PRIMARY								MELANOMA vs. MELANOCYTE			
		Talantov		Kabbarah		Kabbarah		Talantov		Riker		Talantov		Kabbarah		Riker		Kabbarah		Xu		TCGA		Philadelphia		Mannheim	
	Gene_Symbol	Fold change	*p* -value	Fold change	*p* -value	Fold change	*p* -value	Fold change	*p* -value	Fold change	*p* -value	Fold change	*p* -value	Fold change	*p* -value	Fold change	*p* -value	Fold change	*p* -value	Fold change	*p* -value	Fold change	*p* -value	Fold change	*p* -value	Fold change	*p* -value
Effectors	CIT	0.894	0.414	0.966	0.786	1.321	0.014	1.370	0.002	1.560	0.085	1.532	0.000	1.367	0.004	1.009	0.950	1.341	0.000	1.255	0.012	0.914	0.281	1.387	0.051	0.893	0.452
	ILK	2.094	0.009	1.101	0.433	1.099	0.345	3.089	0.000	1.239	0.464	1.475	0.001	0.998	0.983	0.977	0.901	0.906	0.128	0.894	0.141	0.940	0.262	0.663	0.000	1.187	0.071
	LIMK1	0.813	0.465	1.137	0.397	1.059	0.751	1.666	0.002	0.711	0.317	2.049	0.000	0.931	0.665	1.157	0.342	1.173	0.034	1.708	0.000	0.993	0.964	1.190	0.147	1.326	0.003
	LIMK2	0.624	0.056	0.621	0.005	0.666	0.004	0.242	0.000	0.516	0.034	0.388	0.000	1.073	0.542	0.540	0.000	0.661	0.000	0.535	0.000	0.877	0.181	2.029	0.013	4.434	0.000
	MYLK	0.266	0.001	0.531	0.016	0.240	0.000	0.074	0.000	0.526	0.203	0.279	0.000	0.451	0.001	0.594	0.084	0.651	0.001	0.662	0.003	1.647	0.000	2.680	0.034	2.902	0.003
	MYLK3	0.578	0.184	1.037	0.863	1.130	0.434	0.930	0.825	1.312	0.502	1.608	0.041	1.089	0.591	0.870	0.661	1.160	0.049	1.160	0.462	1.347	0.094	0.937	0.707	1.693	0.000
	PAK1	1.611	0.221	0.889	0.669	1.065	0.739	1.395	0.311	0.695	0.499	0.866	0.521	1.197	0.327	1.675	0.032	1.083	0.397	0.924	0.662	1.214	0.040	1.258	0.291	2.346	0.000
	PAK2	0.719	0.020	1.073	0.562	0.949	0.632	0.927	0.474	1.159	0.582	1.289	0.002	0.885	0.225	1.195	0.106	1.273	0.000	1.755	0.000	1.275	0.000	1.372	0.019	2.228	0.000
	PAK3	0.596	0.004	0.860	0.397	0.895	0.565	0.482	0.000	0.645	0.254	0.807	0.040	1.042	0.809	1.039	0.871	1.056	0.603	0.937	0.527	1.647	0.000	1.108	0.472	1.256	0.007
	PAK4	0.837	0.118	1.006	0.960	1.025	0.794	1.182	0.142	0.926	0.792	1.411	0.000	1.019	0.834	1.053	0.722	1.056	0.372	0.981	0.851	0.853	0.032	1.003	0.982	1.121	0.305
	PAK6	1.125	0.597	0.463	0.000	0.387	0.000	0.310	0.000	0.204	0.001	0.275	0.000	0.836	0.215	0.310	0.001	0.541	0.000	0.407	0.000	0.366	0.000	1.536	0.062	2.188	0.000
	PDPK1	3.133	0.000	1.194	0.185	1.166	0.185	4.031	0.000	0.920	0.738	1.287	0.020	0.977	0.812	0.876	0.531	0.862	0.033	0.808	0.009	1.042	0.486	1.512	0.001	2.843	0.000
	PTK2	0.793	0.178	1.066	0.548	1.191	0.355	1.406	0.006	1.320	0.373	1.774	0.000	1.117	0.515	1.173	0.321	1.073	0.517	1.271	0.054	1.057	0.390	1.480	0.006	2.890	0.000
	PTK2B	0.808	0.155	0.695	0.014	0.712	0.095	0.474	0.000	0.597	0.192	0.586	0.000	1.025	0.891	0.866	0.620	0.738	0.005	0.741	0.035	1.357	0.001	0.621	0.000	1.378	0.000
	ROCK1	0.672	0.004	1.245	0.148	1.023	0.861	0.716	0.001	0.975	0.944	1.065	0.332	0.822	0.108	1.205	0.195	1.253	0.004	1.332	0.011	1.647	0.000	1.108	0.577	1.851	0.000
	ROCK2	0.905	0.703	1.183	0.231	1.055	0.596	0.791	0.215	0.809	0.384	0.874	0.350	0.892	0.244	1.005	0.962	1.091	0.075	1.156	0.093	1.670	0.000	1.353	0.015	1.490	0.000

Supplementary table S4. Tabulated data used for the generation of the figure panel reporting on melanin biosynthesis genes in Fig.1b | Fold change for the indicated comparison in the also indicated database is given together with associated p-values. A difference is considered significant if *p* <0.05. If *p* -values are tabulated as "0.000" then they were <0.001.

		NEVUS vs. NORMAL				PRIMARY vs. NORMAL						PRIMARY vs. NEVUS				MET vs. PRIMARY								MELANOMA vs. MELANOCYTE			
		Talantov		Kabbarah		Kabbarah		Talantov		Riker		Talantov		Kabbarah		Riker		Kabbarah		Xu		TCGA		Philadelphia		Mannheim	
	Gene_Symbol	Fold change	*p* -value	Fold change	*p* -value	Fold change	*p* -value	Fold change	*p* -value	Fold change	*p* -value	Fold change	*p* -value	Fold change	*p* -value	Fold change	*p* -value	Fold change	*p* -value	Fold change	*p* -value	Fold change	*p* -value	Fold change	*p* -value	Fold change	*p* -value
Melanin biosynthesis	DCT	16.244	0.000	3.528	0.000	2.435	0.000	2.437	0.083	1.364	0.438	0.150	0.000	0.690	0.029	0.883	0.705	0.735	0.082	0.718	0.105	1.048	0.812	0.809	0.592	1.953	0.042
	SLC45A2	3.720	0.000	1.021	0.953	1.645	0.107	9.749	0.000	9.357	0.034	2.621	0.000	1.611	0.000	0.819	0.658	0.989	0.909	1.217	0.171	0.762	0.108	1.633	0.130	2.618	0.003
	TYR	16.312	0.000	1.873	0.257	2.853	0.033	49.745	0.000	7.756	0.001	3.050	0.000	1.524	0.014	0.793	0.364	0.807	0.143	0.809	0.199	0.653	0.000	0.334	0.008	0.622	0.125
	OCA2	3.060	0.003	0.465	0.499	0.621	0.630	1.384	0.539	3.913	0.111	0.452	0.054	1.336	0.106	0.805	0.676	0.697	0.096	0.713	0.223	0.586	0.206	0.110	0.000	0.092	0.000
	TYRP1	6.023	0.000	2.289	0.000	2.484	0.000	6.877	0.000	1.519	0.237	1.142	0.325	1.085	0.361	0.732	0.356	0.406	0.000	0.426	0.000	0.657	0.068	0.250	0.000	0.555	0.053

Supplementary table S5. Tabulated data used for the generation of the figure panel reporting on control genes in Fig.1b | Fold change for the indicated comparison in the also indicated database is given together with associated p-values. A difference is considered significant if *p* <0.05. If *p* -values are tabulated as "0.000" then they were <0.001.

		NEVUS vs. NORMAL				PRIMARY vs. NORMAL						PRIMARY vs. NEVUS				MET vs. PRIMARY								MELANOMA vs. MELANOCYTE			
		Talantov		Kabbarah		Kabbarah		Talantov		Riker		Talantov		Kabbarah		Riker		Kabbarah		Xu		TCGA		Philadelphia		Mannheim	
	Gene_Symbol	Fold change	*p* -value	Fold change	*p* -value	Fold change	*p* -value	Fold change	*p* -value	Fold change	*p* -value	Fold change	*p* -value	Fold change	*p* -value	Fold change	*p* -value	Fold change	*p* -value	Fold change	*p* -value	Fold change	*p* -value	Fold change	*p* -value	Fold change	*p* -value
Controls	GAPDH	1.644	0.032	1.069	0.644	1.457	0.014	2.269	0.000	1.119	0.291	1.380	0.003	1.363	0.000	0.878	0.155	1.042	0.450	0.851	0.003	0.902	0.221	1.152	0.207	1.419	0.000
	ATP5F1	0.937	0.540	1.071	0.545	0.855	0.086	0.633	0.001	0.972	0.768	0.676	0.001	0.799	0.054	1.095	0.380	1.211	0.003	1.394	0.000	1.084	0.133	1.483	0.006	2.575	0.000
	PGK1	1.087	0.621	0.712	0.078	0.995	0.976	2.643	0.000	1.810	0.029	2.431	0.000	1.399	0.009	1.240	0.229	1.192	0.048	1.592	0.000	1.129	0.032	1.490	0.011	3.000	0.000
	CDH1	1.083	0.690	0.746	0.021	0.780	0.047	1.724	0.000	0.655	0.043	1.592	0.004	1.046	0.652	0.561	0.056	0.583	0.000	0.719	0.050	0.876	0.367	0.109	0.001	0.585	0.170
	CDH2	1.564	0.253	0.818	0.533	1.467	0.220	1.057	0.893	4.409	0.152	0.676	0.255	1.794	0.020	1.582	0.389	1.744	0.018	2.600	0.019	1.815	0.003	7.320	0.000	20.843	0.000

Supplementary Table S6. Tabulated data of SILAC analysis supporting the respective panels in Fig.2 | Maxquant [1] Protein Groups output detailing the identified proteins in the SILAC pulldown experiment and their SILAC ratio values. Contaminants were marked by identifying proteins matching to a database of known contaminants, built into the Maxquant. Perseus [2] outlier test with a *p*-value cut-off of 0.05 (*i.e.* significant if -log(*p*-value)>1.301) was applied to highlight values (green) that have significantly higher H/L ratios relative to the rest of the population.

T: Uniprot	T: Gene Names	M: Ratio H/L Normalized	C: Conta-minant	C: outlier significant	N: -Log outlier sig. *p*-value
B3KNU0;Q4QQI8;P52306-1;P52306;Q9BUX6; B7Z2V8;Q499L7;P52306-2;B7Z4M5;Q6U7G8	RAP1GDS1	27.435		+	323.306
Q92974-2;Q92974;B7Z977;Q5VY93;Q92974-1;Q92974-3;B4DJ49	ARHGEF2;KIAA0651;LFP40;RP11-336K24.3-004	20.379		+	309.957
Q8TEQ6;B7ZLC9;Q58EZ8	GEMIN5	16.22		+	194.291
P23396;Q53G83;Q9NQS8	OK/SW-cl.26;RPS3	15.188		+	169.77
O75116;B4DRJ9;Q14DU5	KIAA0619;ROCK2	14.55		+	155.442
Q16512-2;Q16512;Q16512-1;Q15523;Q504U4	PAK1;PKN;PKN1;PRK1;PRKCL1	12.968		+	122.653
P24752;Q96FG8	ACAT;ACAT1;MAT	11.838		+	101.621
Q16513;B4DQ21;B4DTP5;B4DVG1;Q08AF4;Q6P5W9;B1AL79	PKN2;PRK2;PRKCL2;hCG_23733;RP5-905H16.1-002	10.625		+	81.2605
Q6NX52;Q92888-3;Q92888;Q92888-1;Q92888-2;Q49AN3	ARHGEF1;hCG_22327	8.5136		+	51.2863
P05141;B2RCV1;Q6NVC0	ANT2;SLC25A5	6.1681		+	26.1172
Q8WWN8;A8K1Y7;B3KMD0;B4DIT1;Q05CA7; Q05CH1;Q96G49;Q9H7C1	ARAP3;CENTD3	4.9338		+	16.2972
Q6PFW2;O15085;B7Z7U2;A6NFK4	ARHGEF11;RP11-356J7.2-001;KIAA0380	4.2968		+	12.1488
P12956;B2RDN9;B1AHC8;B1AHC9;B4DE32;B4E356;Q6IC76;B1AHC7	G22P1;XRCC6;CTA-216E10.7-002;hCG_2013151; CTA-216E10.7-005;CTA-216E10.7-004	3.4232		+	7.47145
P13010;Q53T09;Q53TC2	G22P2;XRCC5	3.3456		+	7.11223
P36542-1;P36542;Q6I9V2;Q8TAS0;P36542-2;B4DFE6;B4DL14	ATP5C;ATP5C1;ATP5CL1;hCG_25112;RP11-401E9.1-001	2.4818		+	3.72688

P15880;A4D0Y7;O60249;Q3KQT6;Q6IPX5;Q8J014;Q8N5L9;Q8NI62;Q9BSW5;A6NI39	RPS2;RPS4;LOC392781;tcag7.979;rps2;OK/KNS-cl.6	1.5078			1.2289
P43897-2;P43897;P43897-1;B4E391;B4DHY8;C9JT21	TSFM;hCG_40154	1.412			1.0557
P68104;A8K9C4;A9X7H1;B4DV42;B4E2C5;Q16577;Q53G85;Q53G89;Q53GA1;Q53GE9;Q53HM9;Q53HQ7;Q53HR1;Q53HR5;Q6IPN6;Q6IPS9;Q6IPT9;Q8IUB0;Q96RE1;Q9H2I7;Q9NZS6;Q5VTE0;Q5JR01;A6PW80;Q2F837;Q504Z0;Q6IQ15;Q6P082;Q6P4C9;Q8TBL1;Q96C29;Q96CD8;Q96EB3;B4DNE0;Q05639	EEF1A;EEF1A1;EF1A;LENG7;EEF1A1L14;PTI-1;hCG_2033271;RP11-505P4.2-001;EEF1AL3;RP11-505P4.2-015;RP11-505P4.2-003;EEF1A2;EEF1AL;STN	1.2959			0.86244
P16152;A8MTM1;B4DFK7	CBR;CBR1;CRN;hCG_401084	1.1028			0.580392
P61247;A8K4W0;B7Z3M5;Q6NXR8	FTE1;MFTL;RPS3A	1.0889			0.561945
P21266;B4E2J2;Q59EJ5;Q6FGJ9;A4UJ43	GST5;GSTM3;hCG_40244;RP4-735C1.2-001	1.0678			0.534411
P11021;B4DEF7;Q2KHP4	GRP78;HSPA5	1.0092			0.460876
O00571;A8K538;B4E3E8;B5BTY4;Q59GX6;Q5S4N1;O15523;B4DK29;B4DXX7;B4E010;B4E132	DBX;DDX3;DDX3X;hCG_19318;RP1-169I5.3-001;DBY;DDX3Y	0.91147			0.347675
P09211;C7DJS1;C7DJS2;A8MX94;B2C310	FAEES3;GST3;GSTP1	0.89082			0.325239
P24534;A4D1M6;C9JZW3;C9J741;C9JX93	EEF1B;EEF1B2;EF1B;hCG_19809;LOC392793;tcag7.1190	0.82471			0.256817
P08107;A8K5I0;B4DI39;B4E1S9;Q9UQC1;B4DNT8;B4DWK5;Q59EJ3;B3KTT5;B4DFN9;B4DVU9;B4E388;B4E3B6;Q5SP16;B4DNX1;B4E1T6	HSPA1;HSPA1A;HSPA1B;DAAP-21F2.7-001;DAAP-21F2.8-001;DADB-333F21.2-001;DADB-333F21.4-001;hCG_1820593;hCG_43726;HSP70-1;DAAP-21F2.8-002;DADB-333F21.4-002;DAQB-147D11.2-002;XXbac-BCX40G17.4-002;XXbac-BPG254B15.2-002	0.80237			0.234854
P17066;B2R6X5;B3KSM6;B4DHP5;Q53FC7;P48741;C9IYE6;C9JCM4	HSP70B';HSPA6;HSP70B;HSPA7;HSPA1B	0.79342			0.226217
P11142-1;P11142;A8K7Q2;B3KTV0;B4E1Q1;Q53GZ6;Q	HSC70;HSP73;HSPA10;HSPA8	0.77757			0.211148

96BE0;Q96H53;Q96IS6;Q9NWW3;Q9NZ87;P11142-2;Q53HF2;B4DTX2					
B4DTG2;P26641;Q2F838;Q2F840;Q53YD7;B4DUK7;B4DUP0	EEF1G;EF1G;PRO1608;hCG_2039458	0.74435			0.180493
P13639;B4DMC6;B4DRE8;Q6PK56;Q8TA90;B4DPU3	EEF2;EF2	0.60141			0.0625414
P49327;Q13587;Q6PJJ3	FAS;FASN	0.51887			0.0042997
P38646;B7Z1V7;B7Z4V2;Q2F839;Q8N1C8;A1XP52;B7Z4T3	GRP75;HSPA9;HSPA9B	0.50494			0.00490364
A1E282;Q53GK6;P60709;B4DW52;B7ZAP6;Q1KLZ0;Q53G76;Q53G99;Q562L5;Q562L6;Q562L9;Q562M3;Q562M5;Q562N0;Q562N2;Q562N8;Q562P0;Q562R8;Q562S0;Q562U1;Q562U2;Q562V5;Q562X9;Q562Y6;Q562Y8;Q562Z4;Q562Z6;Q562Z7;Q6PJ43;Q8WVW5;P60712;P63261;B4E3A4;A4UCT3;A5GZ75;B3KWQ3;B4DVQ0;B4E335;Q96DE1;Q96FU6;Q9UE89;Q9UMN3;C9JTH0;Q562N4;Q562P9	ACTB;hCG_15971;PS1TP5BP1;ACT;ACTG1;ACTG	0.49942	+		0.00858744
B4DYH1;Q92598-1;Q92598;B4DF68;Q92598-3;Q92598-2;B4DY72;B4DZB4	hCG_32198;HSPH1;HSP105;HSP110;KIAA0201	0.47454			0.0255721
P08670;B0YJC4;B0YJC5;B3KRK8;Q53HU8	VIM	0.41349			0.0699394
B1AK88;B2R7T8;B4DWA6;P47756-1;P47756;P47756-2;B1AK87;Q32Q68;Q7L4N0;B1AK85;B1AK86;B1AP83;B1AK84;B1AP81	CAPZB;hCG_41078;RP4-657E11.7-008;RP4-657E11.7-001;RP4-657E11.7-002;RP4-657E11.7-009;RP4-657E11.7-005	0.41269			0.0705466
Q5D862	FLG2;IFPS	0.34928	+		0.120848
P02662		0.21503	+		0.241986
P35579-1;P35579;A8K6E4;B4E3S1;Q60FE2;Q86XU5;Q99529;P35579-2;Q2PS10;Q9UMJ0	MYH9;hCG_41454	0.21385			0.243142
Q9P258;A5PLK7	KIAA1470;RCC2;TD60	0.19367			0.263156

Q7Z3M3;C9J2C0;Q9NY65;B3KPW9;B7Z1K5;P68363;A8JZY9;B3KPS3;B3KT06;Q9BQE3;Q53GA7;P68366;A8MUB1;B4DDU2;Q8WU19;A6NHY4;C9JIF5;Q9H853;C9JDS9	DKFZp686L04275;TUBA8;TUBAL2;hCG_21536;TUBA1B;TUBA1C;TUBA6;TUBA1;TUBA4A;hCG_2013418;TUBA4;TUBA4B	0.17941	+		0.277585
A2I7N1;A2I7N0;Q28922;Q3ZEJ6;Q9TTE1	0.13836	+		0.32046	
P34955		0.13176	+		0.32754
P04259	K6B;KRT6B;KRTL1	0.11147			0.349633
Q86YZ3;Q5DT20;Q5W8V9	HRNR;S100A18	0.10975	+		0.351528
P35527	KRT9	0.1038	+		0.358113
P13647;B4DL32;B4E1T1	KRT5	0.093779	+		0.3693
P02533;A2A4G1;Q04695;Q14666	KRT14;KRT17	0.091957	+		0.371347
P81605;Q53YJ2;A5JHP3	AIDD;DCD;DSEP;hCG_1820620;PIF	0.079107			0.385898
P08779;Q16195	KRT16;KRT16A;keratin	0.073409	+		0.392414
Q3SZR3		0.063484	+		0.40386
P02769		0.058628	+		0.409503
P35908	KRT2;KRT2A;KRT2E	0.055278	+		0.413414
P04264	KRT1;KRTA	0.047637	+		0.422384
P13645	KPP;KRT10	0.039093	+		0.4325
P61586;B4DKN9;Q53HM4;Q9BVT0;C9JX21;C9JNR4;P08134;Q5JR08	ARH12;ARHA;RHO12;RHOA;ARH9;ARHC;RHOC;RP11-426L16.4-008	0.034089			0.438466
P00761		0.013005	+		0.463943
O14543;Q5U0H7;Q6FI39	CIS3;SOCS3;SSI3;hCG_1776363	0.011709			0.465527

*****End of supplement*****
